# Force-Induced Shuttling of Rotaxanes Controls Fluorescence Resonance Energy Transfer in Polymer Hydrogels

**DOI:** 10.1021/acsami.2c20904

**Published:** 2023-02-02

**Authors:** Tatsuya Muramatsu, Shohei Shimizu, Jessica M. Clough, Christoph Weder, Yoshimitsu Sagara

**Affiliations:** † Department of Materials Science and Engineering, Tokyo Institute of Technology, Meguro-ku, Tokyo 152-8552, Japan; ‡ Adolphe Merkle Institute, University of Fribourg, Chemin des Verdiers 4, Fribourg CH-1700, Switzerland

**Keywords:** supramolecular mechanophore, rotaxane, FRET, mechanoresponsive luminescence, hydrogel

## Abstract

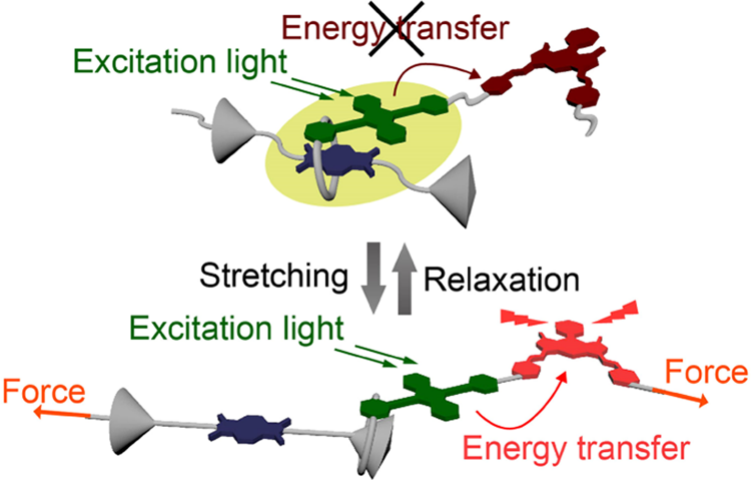

The molecular shuttling function of rotaxanes can be exploited to design mechanoresponsive reporter molecules. Here, we report a new approach to such rotaxane-based mechanophores, in which the fluorescence resonance energy transfer (FRET) between a donor–acceptor pair is mechanically controlled. A cyclic molecule containing a green-light-emitting FRET donor connected to a red-light-emitting FRET acceptor was threaded onto an axle equipped with a quencher at its center and two stoppers in the peripheral positions. In the force-free state, the green emitter is located near the quencher so that charge transfer interactions or photo-induced electron transfer between the two moieties suppress green emission and prevent the FRET from the green to the red emitter. The mechanophore was covalently incorporated into a linear polyurethane-urea (PUU), and stretchable hydrogels were prepared by swelling this polymer with water. Upon deformation of the PUU hydrogels and under an excitation light that selectively excites the donor, the intensity of the red fluorescence increases, as a result of a force-induced separation of the green emitter from the quencher, which enables the FRET. The switching contrast is much more pronounced in the gels than in dry films, which is due to increased molecular mobility and hydrophobic effects in the hydrogel, which both promote the formation of inclusion complexes between the ring containing the green emitter and the quencher.

## Introduction

Mechanochromic responses that are achieved by translating mechanical forces applied to polymeric objects into optical signals allow studying failure and stress-transfer mechanisms and are useful for applications such as pressure sensing, defect monitoring, electronic skins, data encryption, anticounterfeiting features, or tamper-proof packaging materials.^1^ The incorporation of so-called mechanophores—mechanoresponsive motifs—into these materials is one widely employed design approach to achieve such behavior.^2−6^ The usefulness of the rotaxane structure^7−11^ for the design of mechanochromic mechanophores was first recognized by Stoddart and co-workers, who reported that an intramolecular charge transfer complex (CT) was irreversibly dissociated when a tetrahydrofuran (THF) solution of a rotaxane-containing poly(methyl acrylate) was sonicated, leading to the disappearance of the CT absorption.^12^ We showed more recently that the force-induced shuttling function of rotaxanes can be utilized to prepare mechanophores that show instantly reversible changes in photoluminescence intensity.^13−16^ The rotaxane-based supramolecular mechanophores we reported are composed of a cyclic molecule equipped with a fluorophore and an axle molecule containing an electronically matched quencher. When no force is applied, the cycle resides near the quencher and the fluorescence is quenched due to intramolecular CT interactions or photo-induced electron transfer (PeT). The two optically active moieties can be pulled apart by applying a force via two handles attached to the axle and the cycle, respectively, and this is accompanied by a turn-on of the fluorescence. Unless the rotaxane’s stoppers are designed to allow for mechanically induced de-threading,^16^ the process is fully reversible. Thus, rubbery polyurethane elastomers into which the rotaxanes were covalently incorporated display instantly reversible changes of the fluorescence intensity upon being deformed. De Bo and co-workers also reported several rotaxane mechanophores,^17,18^ including a mechanochromic motif in which cycle and axle molecules interact through hydrogen bonds.^19^ The motif was designed to display green fluorescence in the absence of force, due to hydrogen bonding between a fluorophore in the cycle and an aminochloromaleimide (ACM) moiety in the axle molecule. The mechanically induced separation of the cycle and the ACM leads to a significant decrease in fluorescence intensity.

Collectively, the above results suggest that mechanochromic responses should also be attainable in rotaxanes featuring other photofunctional groups, whose assembly and separation promote different photophysical effects. Indeed, here we report a rotaxane-based mechanophore in which a fluorescence resonance energy transfer (FRET) is mechanically switched. FRET effects have been widely used to develop photofunctional materials, including supramolecular polymers,^20−22^ organogels,^23,24^ fluorescent proteins,^25^ and fluorescent probes.^26^ Since the FRET efficiency depends on the distance between donor and acceptor fluorophores and the directions of their transition dipole moments, and because these factors can be controlled by applying mechanical forces, adequately coupled FRET pairs can display mechanochromic behavior.^27−30^ Besides, combination of mechanophore and FRET has been reported to obtain mechanochromic polymer.^31,32^ For our rotaxane mechanophores, bulky fluorophores are not suitable for the emitters directly incorporated into cycle structure because the association constant between the quencher and cycle becomes low, resulting in poor contrast upon stretching the films in which the mechanophores are covalently introduced. Indeed, we previously reported a red-light-emitting supramolecular rotaxane mechanophore with a bulky red fluorophore in the cyclic structure, and the polyurethane film containing the mechanophore exhibited a less pronounced fluorescence contrast.^15^ Introduction of FRET mechanism would make it possible for such nonplanar fluorophores to be involved in rotaxane mechanophores as FRET acceptors without reducing the emission contrast upon stretching because the bulky acceptors don’t need to form π-stacked structure.

**Figure 1:**
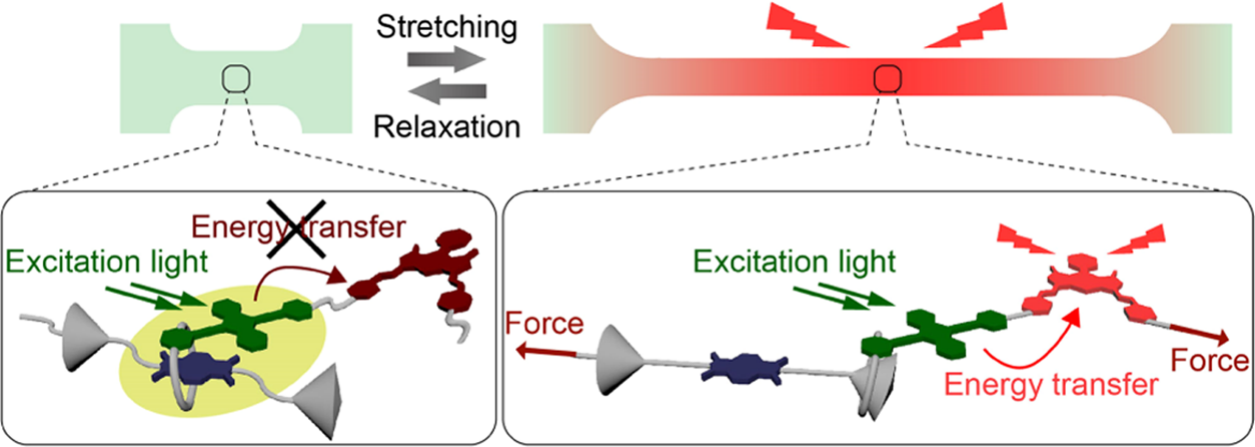
Schematic illustration of FRET control by forces on the rotaxane-based supramolecular mechanophore in stretchable hydrogels. In the force-free state (left), the green emission is quenched because of CT complex formation or PeT.

Although external stimuli such as pH changes,^33,34^ light irradiation,^35^ and changes of solvent polarity^36^ have been used to modulate the efficiency of FRET pairs in rotaxanes, mechanical control over this process has, to our best knowledge, not been demonstrated. The rotaxane mechanophore reported here features a green-light-emitting fluorophore in the cyclic molecule, and this donor is connected via a short spacer to a red acceptor fluorophore (Figure 1). The cycle is threaded onto an axle equipped with a quencher at its center and two stoppers in the peripheral positions. In the force-free state, the emitter is located near the quencher so that the CT interactions or PeT between the two moieties suppress green emission and prevent FRET from the green to red emitter. The mechanophore was covalently incorporated into a linear polyurethane-urea (PUU) and stretchable hydrogels were prepared by swelling with water.^37,38^ Upon deformation of the PUU hydrogels, the intensity of the red fluorescence increases, as a result of the force-induced separation of the emitter from the quencher, which enables the FRET process. Importantly, the ability to tune the emission color of rotaxane mechanophores by simply modifying an existing motif with a FRET acceptor allows one to tune the emission color, which, as we demonstrate, is critical for applications in colored materials.

## Results and Discussion

**Figure 2:**
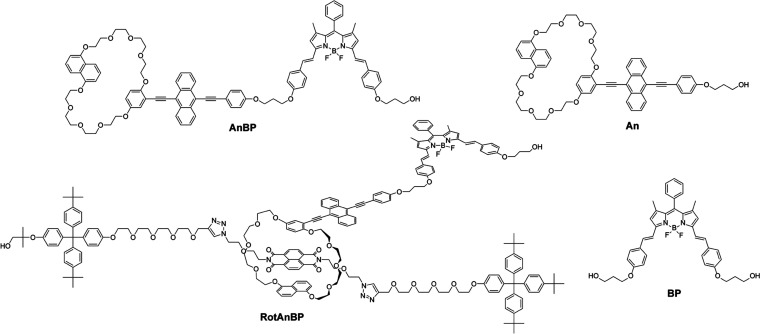
Molecular structures of cyclic compound **AnBP**, rotaxane-based supramolecular mechanophore **RotAnBP**, and reference compounds containing the green (**An**) or red (**BP**) emitter.

**Figure 3:**
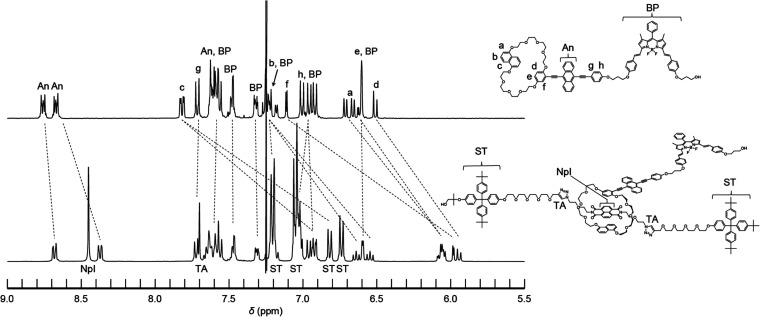
Partial ^1^H NMR spectra of **AnBP** (top) and **RotAnBP** (bottom). The spectra were measured in CDCl_3_ at r.t.

9,10-Bis(phenylethynyl)anthracene was selected as the green fluorophore because of its high fluorescence quantum yield and electron-donating propensity,^14,39−42^ which is pivotal for CT interactions between this fluorophore and 1,4,5,8-naphthalenetetracarboxylic diimide (NpI).^43−45^ NPI was selected as the quencher owing to the electron-deficient character. A π-extended 4,4-difluoro-4-bora-3a,4a-diaza-*s*-indacene (BODIPY) dye, which also displays a high fluorescence quantum efficiency,^46−48^ was chosen as the red fluorophore. The rotaxane **RotAnBP** (Figure 2, left bottom) was designed based on our previously reported rotaxane mechanophores.^13−15^ The cyclic molecule **AnBP** (Figure 2, left top) contains the green emitter and a naphthalene group, which are connected via two tetraethylene glycol chains. The red fluorophore is attached to the green fluorophore through a propyl linker so that efficient energy transfer is possible. The axle features an NpI at its center and two tetraphenylmethane units^49−51^ as stoppers at both ends. **RotAnBP** was prepared through a 1,3-dipolar cycloaddition click-type reaction^52^ between alkyne and azide groups of two precursors of the axle molecule in the presence of a high concentration of **AnBP** (see the Supporting Information for details). The rotaxane mechanophore was characterized by ^1^H NMR and ^13^C NMR spectroscopy and high-resolution electrospray ionization mass spectroscopy. A comparison of the ^1^H NMR spectra of cycle **AnBP** and rotaxane **RotAnBP** shows that diagnostic signals associated with aromatic protons of the cyclic structure and the 9,10-bis(phenylethynyl)anthracene residue are shifted up-field (Figure 3), indicating the formation of an inclusion complex between the cycle and NpI. By contrast, signals ascribed to protons of the π-extended BODIPY hardly change. The rotaxane formation is also evidenced by a decrease in the red fluorescence intensity after rotaxane formation, due to the suppression of FRET (see below). The anthracene derivative **An** (Figure 2, right top) and BODIPY derivative **BP** (Figure 2, right bottom) were also prepared as the reference compounds.

**Figure 4:**
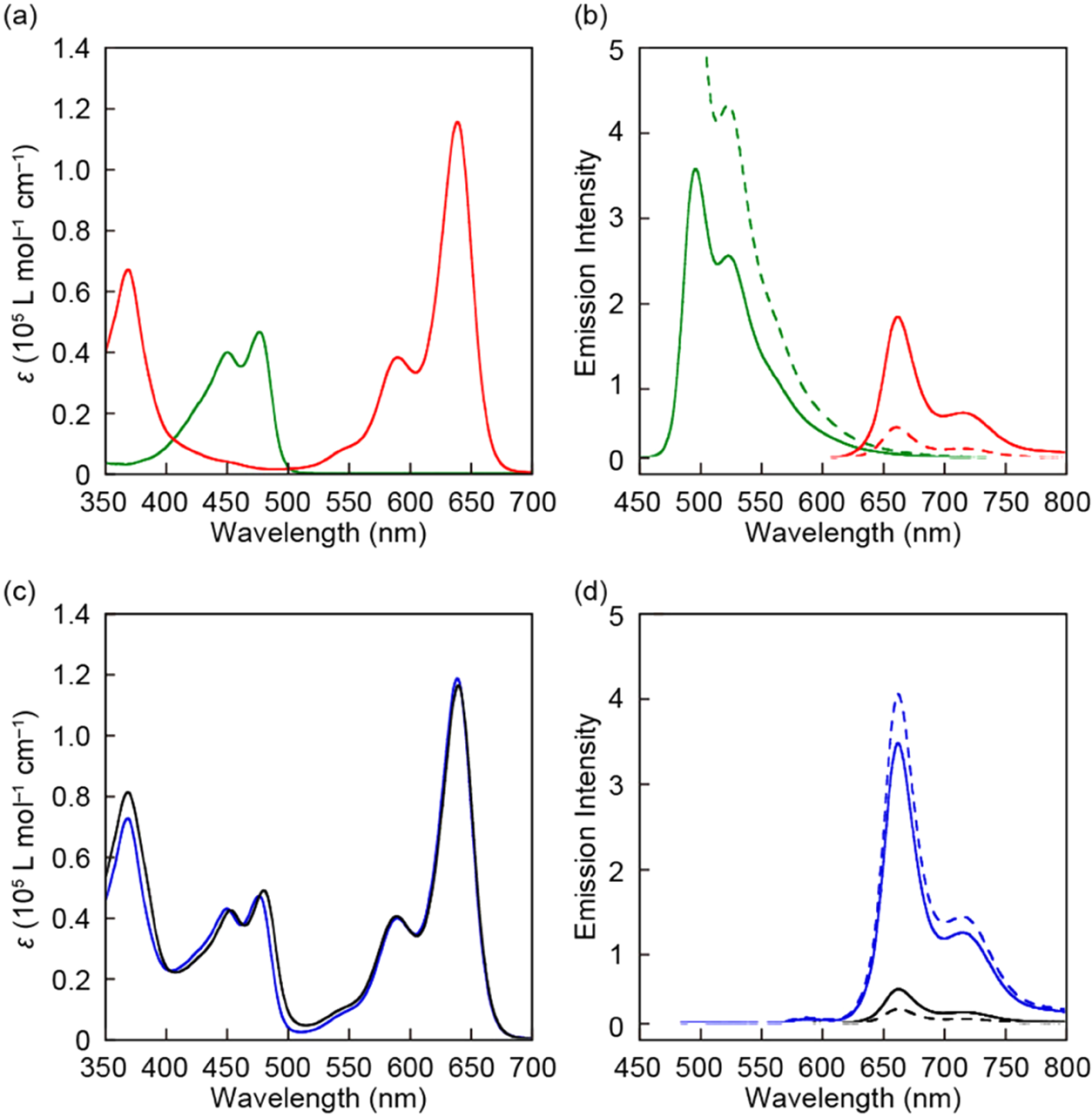
Absorption (a, c) and photoluminescence spectra (b, d) of **An** (green), **BP** (red), **AnBP** (blue), and **RotAnBP** (black) in THF/methanol (1:4, v/v) solutions (*c* = 1 × 10^–5^ M). The photoluminescence spectra were recorded with excitation at 410 nm (solid lines) or 490 nm (dashed lines) at r.t.

The optical characteristics of **An**, **BP**, **AnBP**, and **RotAnBP** were first measured in THF/methanol (1:4, v/v) solutions (*c* = 1 × 10^–5^ M). The absorption spectrum of **An** shows a band with maxima at 450 and 476 nm (Figure 4a, green line). **BP** has one absorption band in the UV region with a maximum at 369 nm and a second band with well-resolved structures and maxima at 590 and 639 nm (Figure 4a, red line). The data show that it is possible to preferentially excite **An** at 490 nm, whereas **BP** can be selectively excited above 500 nm. Under excitation at 410 nm, where the two motifs show a similar extinction (Figure 4a), **An** and **BP** solutions exhibit strong green and red fluorescence, respectively. Both fluorescence spectra show well-resolved peaks with maxima at 495 and 522 nm (**An**, quantum efficiency Φ = 0.84) and 661 and 715 nm (**BP**, Φ = 0.54), respectively (Figure 4b, green and red solid lines). When excited at 490 nm, **BP** shows a much weaker fluorescence than **An** (Figure 4b, green and red dashed lines) because of the lower absorption. By contrast, **An** does not show any fluorescence and **BP** displays strong red fluorescence upon excitation at 590 nm (Figure S1, green and red lines).

The absorption spectrum of **AnBP** shows a superposition of the absorbances of **An** and **BP** (Figure 4c, blue line). However, its fluorescence spectrum displays only the red fluorescence band associated with the BODYPY residue, for both, excitation at 410 and 490 nm (Figure 4d, blue solid and dashed lines), indicating efficient energy transfer from the 9,10-bis(phenylethynyl)anthracene donor to the π-extended BODIPY acceptor. The energy transfer efficiency exceeds 99%, as determined by comparing the intensities of the green fluorescence of solutions of **An** and **AnBP**. The spectral overlap integral of the donor fluorescence and the acceptor absorption and the Förster distance were calculated to be 5.58 × 10^15^ M^–1^ cm^–1^ nm^4^ and 67.1 Å, respectively (see the Supporting Information for details). Based on these values, the energy transfer efficiency is greater than 99% when the distance between the donor and acceptor is within 31.2 Å, supporting that efficient energy transfer occurs in **AnBP**. In a comparison of the red fluorescence intensities of **BP** and **AnBP**, the proportions of the red fluorescence attributed to the direct excitation of the red emitter in **AnBP** are calculated to be *ca.* 50 and 9% under the excitation lights of 410 and 490 nm, respectively. The FRET process is further supported by the fact that the excitation spectrum of **AnBP** recorded at an emission wavelength of 660 nm (Figure S2, blue line) shows a broad band between 400 and 500 nm that corresponds to the absorption band of **AnBP** in the same wavelength region. The overall emission quantum yield Φ_total_ of **AnBP** is 0.54 for excitation at 410 nm, with a yield of below 0.01 for the green emitter (Φ_An_) and 0.54 for the red emitter (Φ_BP_). Moreover, the quantum yield of **AnBP** is hardly affected by the excitation wavelength (Φ_total_ = 0.54, excitation at 490 nm). These results also indicated highly efficient FRET.

The rotaxane **RotAnBP** displays a slightly red-shifted absorption band of the green emitter, due to the CT interaction between the 9,10-bis(phenylethynyl)anthracene and NpI (Figure 4c, black line).^14^ By contrast, the absorption band of the π-extended BODIPY group hardly changes, suggesting that ground-state electronic interactions between the BODIPY moiety and other π-conjugated portions of the rotaxane are absent in solution. As expected, **RotAnBP** exhibits no green fluorescence and only faint red fluorescence in solution when excited at 490 nm (Φ = 0.01, Figure 4d, black dashed line). The excitation spectrum of **RotAnBP** (Figure S2, black line) reveals that the FRET contribution to the red emission is negligible and the faint red emission is caused by direct excitation of the π-extended BODIPY dye, although the absorption of the red emitter at 490 nm is weak. These results reflect that the energy transfer from the green-light-emitting donor to the red-light-emitting acceptor is suppressed, due to the CT interaction or PeT between the donor and the quencher. It is noteworthy that even upon excitation at 410 nm (Figure 4d, black solid line) and 590 nm (Figure S1, black line), the fluorescence intensity of **RotAnBP** is still weaker than that of **BP** and **AnBP** although the BODIPY residue is directly and exclusively excited. The emission quantum yield of the red fluorescence originating from **RotAnBP** is 0.14 when excited at 590 nm, which is much smaller than that of **BP** (Φ = 0.54). Besides, the emission decay of **RotAnBP** monitored at 660 nm in THF/methanol is accelerated relative to that of **BP** and **AnBP** (Figure S3). These results suggest that the BODIPY residue in the rotaxane in the excited state interacts with other π-conjugated groups, leading to dynamic quenching in solution, presumably due to PeT. In contrast to **AnBP**, the quantum yield of **RotAnBP** depends on the excitation wavelength (Φ_BP_ = 0.06, excitation at 410 nm), which supports our conclusion that the FRET is suppressed and red fluorescence is observed only when the BODIPY derivative is directly excited.

Recently, linear PUUs carrying carboxyl groups on the main chain were reported to form hydrogels after swelling with water.^37,38^ The hydrophobic urea segments aggregate to form strong physical cross-links, which bestow the PUU hydrogels with high extensibility and toughness. Speculating that hydrophobic interactions would increase the association constant between the cycle and the quencher, we elected to investigate the mechanoresponsive behavior of **RotAnBP** in hydrogels by integrating the new mechanophore into a hydrophilic PUU. This was achieved by the polyaddition reaction between poly(ethylene glycol) (PEG) (*M*_n_ = *ca*. 2000 g/mol), isophorone diisocyanate, isophorone diamine, and 2,2-bis(hydroxymethyl)propionic acid in the presence of 0.004 mol % of **RotAnBP** (Scheme S1). The reaction was modified from the reported procedures^37,38^ because **RotAnBP** was unstable when heated (see the Supporting Information for details). The resulting polymer **RotAnBP-PUU** is soluble in polar organic solvents, such as methanol, dimethylformamide, and dimethyl sulfoxide and displays a number-average molecular weight of *M*_n_ = 48 kg/mol. The successful incorporation of **RotAnBP** into the polymer was confirmed by the absorption spectrum of **RotAnBP-PUU** in methanol, which shows the diagnostic signals of the chromophores (Figure S4). However, no peaks corresponding to **RotAnBP** can be observed in the ^1^H NMR spectrum of the polymer, on account of its low concentration (Figure S5). The photoluminescence spectrum of a methanol solution of **RotAnBP-PUU** (Figure S4) shows that the FRET process remains suppressed after incorporation into the polymer.

The thermal properties of **RotAnBP-PUU** were investigated by thermogravimetric analysis (TGA) (Figure S6a) and differential scanning calorimetric (DSC) measurement (Figure S6b). The TGA scan shows that decomposition sets in at *ca*. 220 °C, and the DSC trace reveals a weak melting transition at around 15 °C upon heating, which is associated with crystalline PEG domains. These data reflect that the thermal properties of **RotAnBP-PUU** are similar to a similar PUU that was reported previously.^37,38^**RotAnBP-PUU** was processed into thin films with a thickness of 70–100 μm by solvent casting (see the Supporting Information for details). The mechanical properties of the material in the dry state were characterized by dynamic mechanical analysis (DMA) (Figure S7) and uniaxial tensile tests (Figure S8a, Table S1). The DMA traces reveal a glass-transition temperature (*T*_g_) of *ca*. −40 °C, a rubbery plateau that extends to *ca.* 80 °C, and a regime in which the storage modulus decreases with increasing temperature, before the samples fail at *ca.* 180 °C. The tensile tests, which were carried out at r.t., reveal a Young’s modulus of 29.5 ± 7.8 MPa, a tensile strength of 14.5 MPa, and an extensibility of more than 1000%.

**RotAnBP-PUU** hydrogels were prepared by immersing the dried films in deionized water for 2 h; after this time, an equilibrium state with a water take-up of 70% had been reached (Figure S9). Under ambient conditions, the gels slowly dry and water is released with an initial rate of ca. 0.5% min^–1^ (Figure S10). Tensile tests (Figure S8b, Table S1) reveal a Young’s modulus of 1.19 ± 0.04 MPa, which is more than an order of magnitude lower than that of the dry films. The hydrogels display an elongation of *ca.* 1200% and a nominal tensile strength of 5.93 MPa.

The dry **RotAnBP-PUU** films exhibit moderately weak red fluorescence (Φ = 0.05, excited at 490 nm), although the methanol solution of **RotAnBP-PUU** hardly fluoresces under the same excitation conditions. Residual emission in the force-free state was also observed for previously reported polyurethanes containing other rotaxane-based mechanophores,^15^ and appears to be related to residual stresses in the material that cause separation of the green emitter and the quencher in a fraction of the rotaxanes. This enables energy transfer from the excited 9,10-bis(phenylethynyl)anthracene to the π-extended BODIPY. In contrast, the red fluorescence intensity of the **RotAnBP-PUU** hydrogel becomes much weaker (Φ = 0.02, excited at 490 nm) than that of the dried material, likely on account of an increased cycle-quencher association constant due to hydrophobic interactions and the increased molecular mobility, which both should reduce the fraction of activated mechanophores.

**Figure 5:**
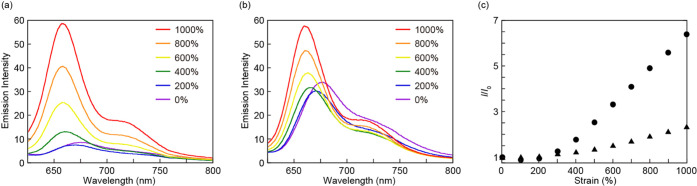
(a, b) Fluorescence spectra of a **RotAnBP-PUU** hydrogel (a) and a dried **RotAnBP-PUU** film (b) recorded upon stretching the samples to the strains indicated. (c) Plots of the relative fluorescence intensity *I*/*I*_0_ of the **RotAnBP-PUU** hydrogel (circles) and the dried **RotAnBP-PUU** film (triangles) as a function of strain. All fluorescence spectra were recorded at r.t. with λ_ex_ = 490 nm. The fluorescence intensities in (c) were recorded at 660 nm.

The force-induced shuttling function controls the on/off switching of FRET, as depicted in Figure 1. Upon uniaxial tensile deformation, the **RotAnBP-PUU** hydrogel initially shows a slight decrease in the red fluorescence intensity due to a decrease in the thickness of the hydrogel, and then exhibits subsequently a considerable increase in the red fluorescence intensity when excited at 490 nm (Figure 5a, Figure S11a, Movies S1). Upon subsequent removal of the force, the fluorescence intensity gradually decreases (Figure S12). This indicates that the increase in the red fluorescence intensity of the hydrogel with excitation at 490 nm is mainly due to the activation of the FRET process. To the best of our knowledge, this is the first report on the on/off switching of the FRET by force on rotaxanes at single molecular level. Even upon excitation at 590 nm, the red fluorescence intensity of the hydrogels increases; however, the attainable contrast or relative increase is smaller than that achieved when exciting the sample at 490 nm (Figure S13). The fluorescence contrast between unstretched and stretched states is improved compared to our previously reported red-emitting rotaxane, in which a BODIPY-based red emitter was directly introduced into cyclic moiety.^15^

From a practical viewpoint, the rotaxane **RotAnBP** functions as a red-emissive supramolecular mechanophore in hydrogels. Red luminescence is useful for multicolor imaging and for developing white-emissive materials. Besides red emission can penetrate into the living tissues deeply, making it favorable for bioimaging. Thus, the development of the red-light-emitting mechanophores is desirable to expand the application of mechanophores. The possibility to readily vary the emission color of a given mechanophore is further useful for applications in colored materials. To demonstrate this, we carried out experiments in which we covered the **RotAnBP-PUU** hydrogel with a polyurethane film containing a red dye (see Figures S14–S19 and the Supporting Information for details). Gratifyingly, a clear increase in the red fluorescence intensity was observed upon stretching the **RotAnBP-PUU** hydrogel whether or not the red film cutting light of the wavelength ranging below 600 nm was applied (Figure S18). By contrast, when a reference polyurethane film containing the “parent”, green-light-emitting rotaxane mechanophore without the FRET acceptor^16^ was stretched, virtually all of the green fluorescence was absorbed by the red dye (Figure S19).

The dry **RotAnBP-PUU** films also show a mechanoresponse upon stretching (Figure 5b, Figure S11b, Movies S2). However, plots of the relative fluorescence intensities, i.e., the ratio of the fluorescence intensities of the stretched (*I*) and unstretched (*I*_0_) samples recorded at 660 nm (Figure 5c) show that the contrast observed for the **RotAnBP-PUU** hydrogels is much larger than that in the dry **RotAnBP-PUU** films, although significantly higher stresses are required to deform the dry films (Figure S8). The difference is caused by the weak fluorescence intensity of the hydrogel in the force-free state and perhaps also a lower fraction of activated mechanophores in the material because of the bulky structure of the ring featuring the FRET pair.

## Conclusions

In conclusion, we successfully demonstrated that the mechanically induced molecular shuttling function of rotaxanes can be used to control the on/off switching of a FRET process between a donor–acceptor pair in the reporter molecule. The FRET pair, a green-emissive energy donor and a red-emissive energy acceptor, were connected via a short spacer and attached to the cyclic part of the rotaxane. The photophysical properties of the fluorophores allow the preferred (although not exclusive) excitation of the donor at 490 nm. An efficient FRET process causes the cyclic molecule to emit almost exclusively red light, even if the donor is preferentially excited. Upon rotaxane formation, CT interactions or PeT between the green emitter and NpI residue placed in the axle prevent the FRET so that the red fluorescence becomes weak if the donor is excited. The new rotaxane mechanophore was covalently incorporated into a PUU, which was swelled with water to produce a PUU hydrogel. The hydrogel displays an up to 7-fold increase of the red emission intensity upon mechanical deformation. The contrast between the idle and the mechanically activated state is considerably higher than that observed for the dry polymer, on account of the increased association constant between the cycle and NpI as well as the higher molecular mobility.

The results of the present study suggest that rotaxane mechanophores can be used to control photophysical processes that go beyond FRET, for example, circularly polarized emission, phosphorescence, and so on, allowing access to fascinating and sophisticated supramolecular mechanophores. Furthermore, the data suggest that supramolecular mechanophores may function better in hydrophilic environments and that increasing the molecular mobility improves the supramolecular mechanophore property.

## Experimental Section

### Materials

All reagents and solvents were purchased from Merck, Kanto Chemical, Tokyo Kasei, or FUJIFILM Wako Pure Chemical Corporation. Anhydrous dimethylacetamide (DMAc) and methanol were used as solvents for the synthesis of polymers and solvent casting, respectively. Telechelic poly(ethylene glycol) (*M*_n_ = 2000 g/mol) and 2,2-bis(hydroxymethyl)propionic acid were dried in vacuo at 100 °C for 1 h before use. Isophorone diisocyanate was distilled under a vacuum and stored over molecular sieves at 4 °C. Isophorone diamine was dried over molecular sieves at r.t. for 1 day before use. Deionized water for hydrogel preparation was obtained using a Merck Direct-Q UV5.

### Synthesis Procedure

The detailed synthesis procedures to prepare compounds are shown in the Supporting Information (Schemes S1–S4).

### Synthesis of Polyurethane-Urea **RotAnBP-PUU**

Dibutyltin dilaurate (2 drops) was added to a stirred mixture of **RotAnBP** (6.0 mg, 1.7 μmol), telechelic hydroxy-terminated poly(ethylene glycol) (*M*_n_= 2000 g/mol, 1.0 g, 0.50 mmol), isophorone diisocyanate (167 mg, 0.750 mmol), and 2,2-bis(hydroxymethyl)propionic acid (33.5 mg, 0.250 mmol) in DMAc (4 mL) and the mixture was stirred at r.t. for 6 h (Scheme S5). A solution of isophorone diisocyanate (383 mg, 1.72 mmol) in DMAc (2 mL) was then added. After the reaction mixture was stirred at r.t. for an additional 12 h, a solution of isophorone diamine (255 mg, 1.50 mmol) in DMAc (4 mL) was added dropwise over the course of 2 h and the reaction mixture was stirred at r.t. for another 24 h. Methanol (10 mL) was added, and the reaction mixture was stirred for 30 min. Then, the reaction mixture was poured into a mixture of hexane (400 mL) and ethyl acetate (800 mL). The green precipitate was collected by filtration and dried in vacuo for 12 h at 40 °C to afford **RotAnBP-PUU** as a green rubbery solid (1.70 g, 92%, *M*_n_ = 48 kg/mol, PDI = 2.04).

### Preparation of Dried PUU Films and PUU Hydrogels

**RotAnBP-PUU** (400 mg) was dissolved in methanol (8 mL), and the solution was divided between two square poly(tetrafluoroethylene) molds (51 × 51 × 5.0 mm^3^). The molds were placed under an inverted funnel to control the evaporation rate. The solvent was evaporated over the course of 12 h under ambient conditions, and the resulting films were further dried in vacuo at 40 °C for 6 h. The green films thus obtained were smooth and transparent with a thickness of 70–100 μm. After fully swelling the films in a large amount of deionized water for 2 h, transparent hydrogels were obtained with a thickness of 110–150 μm.

## Supporting Information Available

Experimental procedures and additional data (PDF)
Response of the **RotAnBP-PUU** hydrogel to mechanical stress (MP4)
Response of the dried **RotAnBP-PUU** film to mechanical stress (MP4)
