# Supplementary material for: Force-Induced Shuttling of Rotaxanes Controls Fluorescence Resonance Energy Transfer in Polymer Hydrogels
Source: ACS Appl Mater Interfaces. 2023 Feb 2;15(6):8502–9. doi: 10.1021/acsami.2c20904 (PMC9940108; doi:10.1021/acsami.2c20904)
Supplement: Supplementary file 1 — am2c20904_si_001.pdf [file am2c20904_si_001.pdf]

Supporting Information

Force-induced Shuttling of Rotaxanes Controls Fluorescence Resonance Energy Transfer in Polymer Hydrogels

Tatsuya Muramatsu,† Shohei Shimizu,† Jessica M. Clough,‡ Christoph Weder,‡,* and Yoshimitsu Sagara†,*

† Department of Materials Science and Engineering, Tokyo Institute of Technology, Meguro-ku, Tokyo 152-8552, Japan
‡ Adolphe Merkle Institute, University of Fribourg, Chemin des Verdiers 4, CH-1700 Fribourg, Switzerland

E-mail: christoph.weder@unifr.ch; sagara.y.aa@m.titech.ac.jp

Table of Contents

**General Methods**	**S2**
**Synthesis of a Cyclic Compound and a Rotaxane**	**S3**
**Polymer Synthesis**	**S6**
**Fluorescence Spectra of An, BP, AnBP, and RotAnBP with Excitation at 590 nm**	**S7**
**Calculation of Spectral Overlap Integral and Förster distance**	**S7**
**Excitation Spectra of AnBP and RotAnBP in THF/methanol**	**S8**
**Time-Resolved Fluorescence Spectroscopy for BP, AnBP, and RotAnBP**	**S8**
**Absorption and Fluorescence Spectra of RotAnBP-PUU**	**S9**
**^{1}H NMR Spectrum of RotAnBP-PUU**	**S9**
**Thermal Properties of Dried RotAnBP-PUU Films**	**S10**
**Mechanical Properties of Dried RotAnBP-PUU Films and RotAnBP-PUU Hydrogels**	**S10**
**Change of Water Content upon Swelling the Dried RotAnBP-PUU Film**	**S12**
**Decrease of Water Content in RotAnBP-PUU Hydrogel**	**S12**
**Images of RotAnBP-PUU Hydrogel and Dried RotAnBP-PUU Films upon Uniaxial Deformation**	**S13**
**Changes in the Fluorescence Intensity of RotAnBP-PUU Hydrogel upon Relaxation**	**S13**
**Changes in the Fluorescence Intensity of RotAnBP-PUU Hydrogel upon Excitation at 590 nm**	**S14**
**Examination of the Penetration of the Red Fluorescence Emitted by RotAnBP-PUU Hydrogels**	**S14**
**References**	**S17**
**Supporting Movies**	**S17**
**NMR Spectra**	**S18**

General Methods

All reactions were performed under nitrogen atmosphere, unless otherwise noted. Flash silica gel column chromatography was carried out with a Biotage Isolela Flash system using SHOKO-scientific Purif-Pack-EX cartridges. Silica gel from Kanto Chemicals (silica gel 60 N, spherical, 40–50 μm) was used for conventional silica gel column chromatography. Recycling preparative gel permeation chromatography (GPC) was conducted with a Japan Analytical Industry LaboACE.

^{1}H NMR spectra were acquired on a JEOL JNM-ECX 400 spectrometer or a JEOL JNM-ECZ400S/L1 spectrometer and all chemical shifts are reported on the δ-scale in ppm relative to the signal of tetramethylsilane (at 0.00 ppm) as an internal standard. Coupling constants (J) are quoted in Hz and relative intensities are also shown. Proton-decoupled ^{13}C NMR spectra were conducted with a JEOL JNM-ECX 400 spectrometer or a JEOL JNM-ECZ400S/L1 spectrometer and all chemical shifts are expressed in ppm using the solvent as an internal standard ($CDCl_3$ at 77.16 ppm or DMSO-d_6 at 39.52 ppm).

Matrix-assisted laser desorption ionization time-of-flight (MALDI-TOF) mass spectra were measured on an AB SCIEX TOF/TOF 5800 system or a SHIMAZHU AXIMA-performance. High-resolution electrospray ionization (ESI) mass spectroscopy was performed with a Bruker Daltonics micrOTOF II.

Size-exclusion chromatography experiments were performed on an Agilent 1260 Infinity II HPLC system equipped with one Agilent PolarGel M guard column (particle size = 8 μm) and two Agilent PolarGel M columns (ID = 7.5 mm, L = 300 mm, particle size = 8 μm). Signals were recorded by a UV detector (Agilent 1260 series) and an interferometric refractometer (Agilent 1260 series). Samples were run using DMF + 0.05 M LiBr as the eluent at 60 °C and a flow rate of 1.0 mL/min. Molecular weights were determined based on narrow molecular weight poly(ethylene oxide) calibration standards.

Thermogravimetric analysis (TGA) was performed under nitrogen with a Mettler-Toledo Star^{e} system at a rate of 10 °C/min. Differential scanning calorimetry (DSC) measurements were conducted under N_2 on a Mettler Toledo DSC 2 STAR system at heating and cooling rates of 10 °C/min. Dynamic mechanical analyses (DMA) were carried out under N_2 with a TA Instruments DMA Q800 at a heating rate of 3 °C/min, a frequency of 1 Hz, and an amplitude of 15 μm. Stress-strain measurements were conducted under ambient conditions with a SHIMADZU AGS-100NX equipped with a 100 N load cell at a strain rate of 60 mm/min.

Absorption spectra of solutions were measured on a JASCO V-750. Steady-state fluorescence spectra of solutions were recorded with a JASCO FP-6500 and the spectra were corrected for the detector nonlinearity. The slit width at all excitation wavelengths was 3 nm. Excitation spectra were also recorded with the same fluorescence spectrometer. Steady-state fluorescence spectra of polyurethane-urea films and hydrogels during stretching experiments using a SHIMADZU AGS-100NX equipped with a 100 N load cell were monitored with an Ocean Insight QEPro-FL equipped with a Reflection/Backscattering Prove R400-7-UV-Vis; these spectra were not corrected. The excitation lights at 490 and 590 nm were obtained by passing the light of an Asahi Spectra CL-1501 equipped with an Asahi Spectra CL-H1-505-9-1 or CL-H1-590-9-1 through a respective bandpass filter (Asahi Spectra HMX490 or HMX590). The excitation light at 365 nm was obtained using an Ocean Insight LLS-365 LED light source. Photographs and movies were taken with a Canon EOS 9000D stabilized with a tripod.

Synthesis of a Cyclic Compound and a Rotaxane

4,4-Difluoro-1,3,5,7-tetramethyl-8-phenyl-4-bora-3a,4a-diaza-*s*-indacene and compounds **An**, **4**, and **5** were synthesized following previous reports.[S1–S3]

Scheme S1. Synthesis scheme of compound **1**.

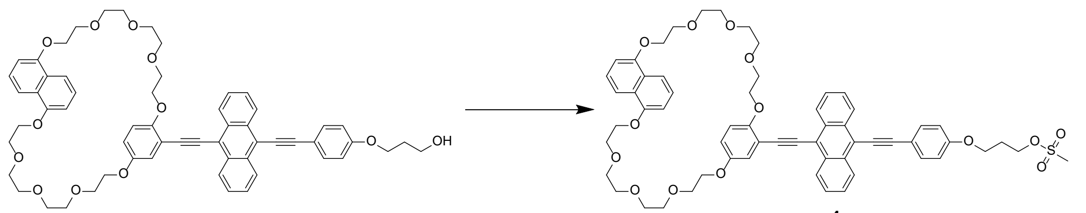

An **1**

Conditions: methanesulfonyl chloride, Et_3N, CH_2Cl_2, r.t., 4 h.

Compound 1. Methanesulfonyl chloride (32 mg, 0.28 mmol) was added to a mixture of compound **An**[S1] (180 mg, 0.187 mmol) and Et_3N (189 mg, 1.87 mmol) in dichloromethane (50 mL) and the mixture was stirred for 4 h at r.t. The reaction mixture was poured into dichloromethane (50 mL), and the solution was washed with 5% aq. HCl (50 mL), saturated aq. $NaHCO_3$ (100 mL), and saturated aq. NaCl (100 mL). The organic layer was dried over $MgSO_4$ and filtered, and the solvent was evaporated. The crude product thus isolated was purified by flash column chromatography on silica gel (eluent: gradient from dichloromethane/acetone = 19:1 v/v to dichloromethane/acetone = 4:1 v/v) to afford compound **1** (180 mg, 0.173 mmol, 93%) as a yellow solid.

^{1}H NMR (400 MHz, $CDCl_3$): δ = 2.20–2.26 (m, 2H), 2.99 (s, 3H), 3.61–3.65 (m, 6H), 3.72–3.75 (m, 8H), 3.78–3.81 (m, 4H), 3.83–3.88 (m, 4H), 3.98–4.00 (m, 6H), 4.08–4.12 (m, 4H), 4.18–4.21 (m, 2H), 4.42–4.45 (m, 2H), 6.49 (d, J = 8.8 Hz, 1H), 6.60 (dd, J = 8.8, 2.8 Hz, 1H), 6.65–6.71 (m, 2H), 6,94 (d, J = 8.8 Hz, 2H), 7.12 (d, J = 2.8 Hz, 1H), 7.22–7.27 (m, 2H), 7.61–7.65 (m, 4H), 7.69 (d, J = 8.4 Hz, 2H), 7.81–7.84 (m, 2H), 8.65–8.69 (m, 4H), 8.75–8.79 (m, 2H).

^{13}C NMR (100 MHz, $CDCl_3$): δ = 29.10, 37.30, 63.37, 66.70, 67.89, 68.04, 68.51, 69.68, 69.75, 69.82, 70.81, 70.95, 71.00, 71.02, 85.67, 90.79, 99.39, 102.44, 105.58, 113.09, 113.35, 114.50, 114.67, 114.72, 116.04, 116.58, 118.46, 118.73, 125.18, 125.22, 126.68, 126.78, 127.21, 127.70, 131.98, 132.04, 133.31, 152.51, 154.02, 154.33, 154.34, 158.89.

MS (MALDI-TOF): m/z: 1060.78 (calcd. $[M+H]^+$ = 1061.38).

Scheme S2. Synthesis scheme of compounds **2**, **3**, and **BP**.

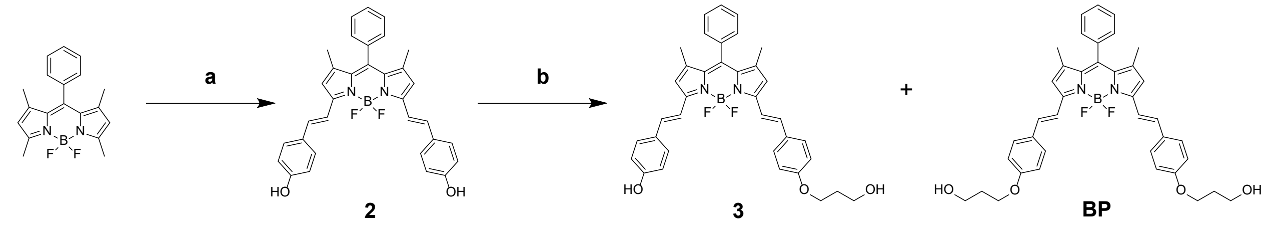

2 **3** **BP**

Conditions: (a) 4-hydroxybenzaldehyde, piperidine, acetic acid, toluene, reflux, 6 h; (b) 3-bromopropanol, K_2CO_3, NaI, acetone, reflux, 24 h.

Compound 2. A mixture of 4,4-difluoro-1,3,5,7-tetramethyl-8-phenyl-4-bora-3a,4a-diaza-*s*-indacene[S2] (600 mg, 1.85 mmol), 4-hydroxybenzaldehyde (422 mg, 3.70 mmol), piperidine (1.2 mL), and acetic acid (1.0 mL) in toluene (50 mL) was stirred for 6 h under reflux using a Dean-Stark trap. After cooling to r.t., the reaction mixture was poured into ethyl acetate (150 mL). The solution was washed with 5% aq. HCl (50 mL), saturated aq. $NaHCO_3$ (100 mL), and saturated aq. NaCl (100 mL). The organic layer was dried over $MgSO_4$ and filtered, and the solvent was

evaporated. The crude product thus isolated was purified by flash column chromatography on silica gel (eluent: gradient from hexane/ethyl acetate = 4:1 v/v to hexane/ethyl acetate = 1:3 v/v) and subsequently precipitated from a mixture of acetone and hexane to afford compound **2** (607 mg, 1.14 mmol, 62 %) as a dark red solid.
^{1}H NMR (400 MHz, DMSO-d_6): δ = 1.40 (s, 6H), 6.85–6.92 (m, 6H), 7.34 (d, J = 16.0 Hz, 2H), 7.42–7.50 (m, 8H), 7.56–7.61 (m, 3H).
^{13}C NMR (100 MHz, DMSO-d_6): δ = 14.22, 114.98, 116.12, 117.96, 127.32, 128.41, 129.06, 129.18, 132.33, 134.36, 137.06, 137.64, 141.23, 152.26, 159.06.
MS (MALDI-TOF): m/z: 555.66 (calcd. [M]$^+$ = 555.20).

Compounds 3 and **BP.** A mixture of compound **2** (500 mg, 0.939 mmol), 3-bromopropanol (109 mg, 0.783 mmol), and K_2CO_3 (325 mg, 2.35 mmol) in acetone (100 mL) was stirred for 24 h under reflux. After cooling to r.t., the reaction mixture was poured into ethyl acetate (150 mL). The solution was washed with saturated aq. NaCl (2 × 100 mL). The organic layer was dried over $MgSO_4$ and filtered, and the solvent was evaporated. The crude product thus isolated was purified by flash column chromatography on silica gel (eluent: gradient from dichloromethane/acetone = 19:1 v/v to dichloromethane/acetone = 4:1 v/v) and subsequently precipitated from a mixture of acetone and hexane to afford compound **3** (53 mg, 0.090 mmol, 11%) as a dark blue solid and compound **BP** (20 mg, 0.031 mmol, 4%) as a dark blue solid.
Compound 3: ^{1}H NMR (400 MHz, $CDCl_3$): δ = 1.44 (s, 6H), 1.71 (t, J = 5.2 Hz, 1H), 2.06–2.12 (m, 2H), 3.88–3.92 (m, 2H), 4.18 (t, J = 6.0 Hz, 2H), 5.01 (s, 1H), 6.62 (s, 2H), 6.87 (d, J = 8.4 Hz, 2H), 6.94 (d, J = 8.8 Hz, 2H), 7.18–7.23 (m, 2H), 7.32–7.34 (m, 2H), 7.48–7.63 (m, 9H).
^{13}C NMR (100 MHz, DMSO-d_6): δ = 14.20, 14.24, 32.08, 57.24, 64.79, 114.96, 115.12, 115.90, 116.13, 117.92, 118.12, 127.31, 128.37, 128.75, 128.82, 129.14, 129.18, 132.31, 132.51, 134.33, 136.33, 137.38, 137.85, 141.09, 141.61, 151.77, 152.69, 159.12, 159.81.
MS (MALDI-TOF): m/z: 590.29 (calcd. [M]$^+$ = 590.26).
Compound BP: ^{1}H NMR (400 MHz, $CDCl_3$): δ = 1.43 (s, 6H), 1.74 (t, J = 5.2 Hz, 2H), 2.05–2.11 (m, 4H), 3.88–3.92 (m, 4H), 4.18 (t, J = 6.0 Hz, 4H), 6.62 (s, 2H), 9.94 (d, J = 8.4 Hz, 4H), 7.21 (d, J = 16.0 Hz, 2H), 7.32–7.34 (m, 2H), 7.49–7.52 (m, 3H), 7.57–7.63 (m, 6H).
^{13}C NMR (100 MHz, $CDCl_3$): δ =14.76, 32.06, 60.51, 65.85, 114.88, 117.37, 117.63, 128.63, 129.02, 129.19, 129.79, 133.29, 135.38, 135.83, 138.26, 141.95, 152.77, 159.69.
MS (MALDI-TOF): m/z: 649.09 (calcd. [M]$^+$ = 649.30).

Scheme S3. Synthesis scheme of cycle **AnBP**.

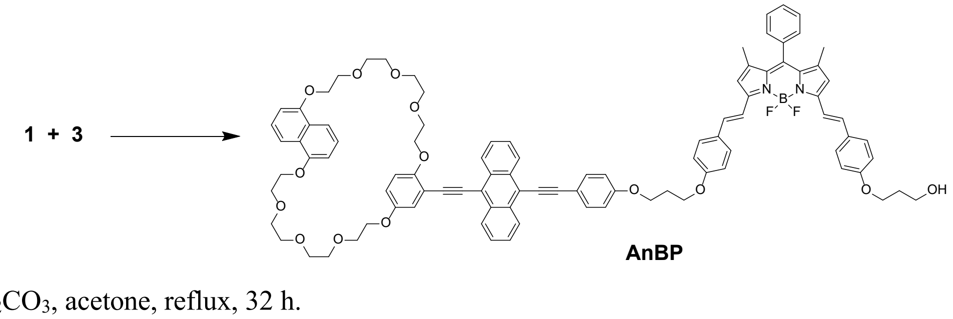

Conditions: K_2CO_3, acetone, reflux, 32 h.

Compound AnBP. A mixture of compound **1** (520 mg, 0.948 mmol), compound **3** (131 mg, 0.942 mmol), and K_2CO_3 (400 mg, 2.89 mmol) in acetone (50 mL) was stirred for 32 h under reflux. After cooling to r.t., the reaction mixture was poured into ethyl acetate (150 mL). The solution was washed with saturated aq. NaCl (2 × 100 mL). The organic layer was dried over $MgSO_4$ and filtered, and the solvent was evaporated. The crude product thus isolated was purified

by flash column chromatography on silica gel (eluent: gradient from dichloromethane/acetone = 9:1 v/v to dichloromethane/acetone = 3:1 v/v) and recycling GPC (eluent: chloroform) to afford compound **AnBP** (167 mg, 0.283 mmol, 29%) as a dark green solid.

^{1}H NMR (400 MHz, $CDCl_3$): δ = 1.42 (s, 6H), 2.02–2.08 (m, 2H), 2.30–2.36 (m, 2H), 3.62–3.66 (m, 6H), 3.73–3.90 (m, 18H), 3.97–4.02 (m, 6H), 4.10–4.16 (m, 4H), 4.20–4.26 (m, 6H), 6.52 (d, J = 9.2 Hz, 1H), 6.61–6.64 (m, 3H), 6.66–6.73 (m, 2H), 6.92 (d, J = 8.8 Hz, 2H), 6.96 (d, J = 8.8 Hz, 2H), 7.01 (d, J = 9.2 Hz, 2H), 7.12 (d, J = 2.8 Hz, 1H), 7.18–7.27 (m, 4H), 7.31–7.34 (m, 2H), 7.47–7.50 (m, 3H), 7.55–7.65 (m, 10H), 7.72 (d, J = 8.8 Hz, 2H), 7.82–7.84 (m, 2H), 8.67–8.69 (m, 2H), 8.76–8.79 (m, 2H).

^{13}C NMR (100 MHz, $CDCl_3$): δ = 14.74, 29.31, 32.03, 60.38, 64.54, 64.66, 65.76, 67.94, 68.10, 68.60, 69.74, 69.81, 69.88, 70.88, 71.00, 71.07, 85.58, 90.88, 99.33, 102.70, 105.63, 113.18, 113.47, 114.55, 114.73, 114.88, 115.74, 116.64, 117.32, 117.62, 118.49, 118.67, 125.22, 125.26, 126.74, 126.80, 127.31, 127.72, 128.60, 129.15, 129.72, 132.02, 132.10, 132.26, 133.33, 135.33, 135.84, 138.21, 141.90, 152.57, 152.75, 154.07, 154.38, 159.37, 159.68, 159.72.

HRMS (ESI): m/z: 1555.6429 (calcd. $[M+Na]^+$ = 1555.6441).

Scheme S4. Synthesis scheme of rotaxane **RotAnBP**.

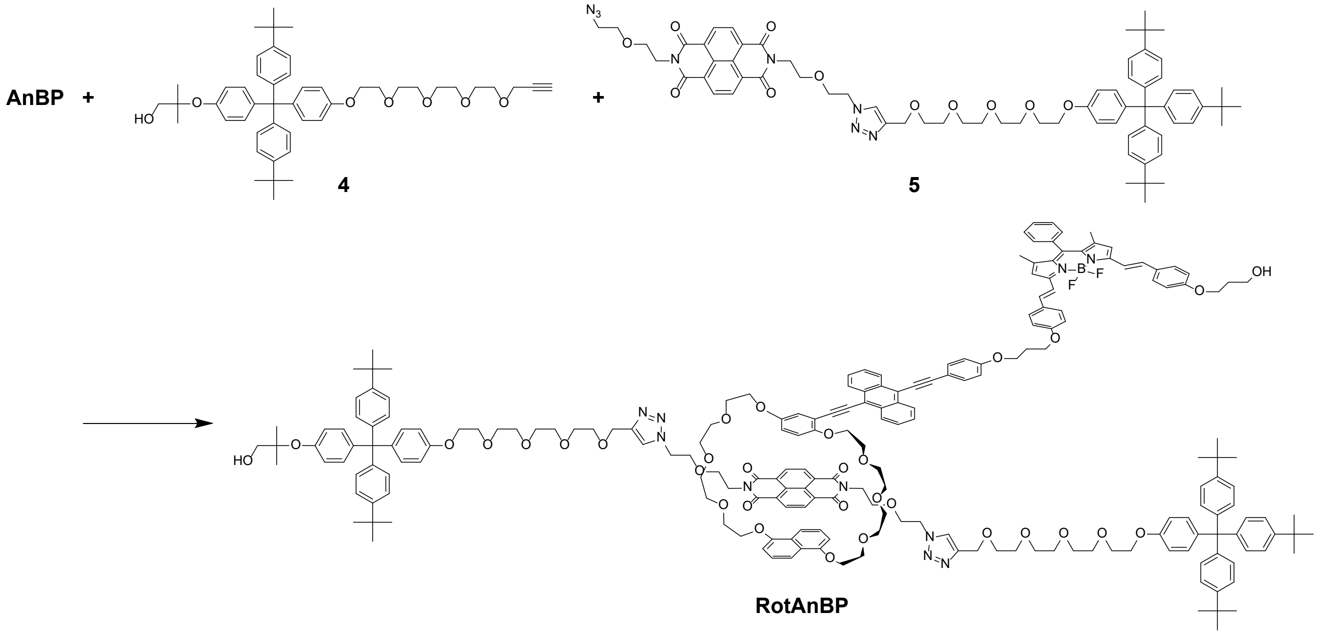

Conditions: $CuSO_4$, sodium ascorbate, $CHCl_3$, H_2O, 5 °C, 15 h.

Compound RotAnBP. A mixture of sodium ascorbate (59.4 mg, 0.300 mmol) and copper (II) sulfate (23.9 mg, 0.150 mmol) in water (1 mL) was added to a solution of compound **AnBP** (153 mg, 0.100 mmol), compound **4**[S3] (73.5 mg, 0.100 mmol), and compound **5**[S3] (121 mg, 0.100 mmol) in chloroform (0.5 mL) and the mixture was vigorously stirred for 15 h at 5 °C. The suspension was poured into a mixture of water (100 mL) and chloroform (150 mL). The organic layer was separated off, washed with saturated aq. NaCl (100 mL), dried over $MgSO_4$, and filtered. After the solvent was evaporated, the crude product thus isolated was purified by flash column chromatography on silica gel (eluent: gradient from dichloromethane/acetone = 4:1 v/v to dichloromethane/acetone = 2:3 v/v) and recycling GPC (eluent: chloroform) to afford **RotAnBP** (30.3 mg, 8.66×10^{-3} mmol, 9%) as a dark green solid.

^{1}H NMR (400 MHz, $CDCl_3$): δ = 1.27 (s, 6H), 1.29 (s, 45H), 1.43 (s, 6H), 2.00 (br, 1H), 2.04–2.08 (m, 2H), 2.26 (t, J = 6.4 Hz, 1H), 2.35 (quin, J = 6.0 Hz, 2H), 3.46–4.08 (m, 80H), 4.17 (t, J = 6.0 Hz, 2H), 4.24–4.29 (m, 4H), 4.45–4.47 (m, 4H), 4.68 (s, 4H), 5.96 (d, J = 9.2 Hz, 1H), 6.00 (d, J = 3.2 Hz, 1H), 6.05–6.19 (m, 3H), 6.56–6.67 (m, 4H),

6.75 (d, J = 8.8 Hz, 4H), 6.82–6.84 (m, 3H), 6.92–7.08 (m, 23H), 7.31–7.34 (m, 2H), 7.48–7.50 (m, 3H), 7.56–7.75 (m, 14 H), 8.38–8.40 (m, 2H), 8.47 (s, 4H), 8.68–8.70 (m, 2H).
^{13}C NMR (100 MHz, $CDCl_3$): δ = 14.74, 23.27, 29.33, 31.50, 32.09, 34.41, 38.50, 50.43, 60.29, 63.04, 63.12, 64.57, 64.65, 65.76, 67.25, 67.45, 67.59, 67.70, 68.66, 69.55, 69.77, 69.84, 69.99, 70.07, 70.47, 70.60, 70.67, 70.84, 71.09, 71.18, 71.29, 71.40, 71.45, 80.54, 85.53, 90.44, 98.26, 103.08, 103.57, 103.61, 110.12, 111.69, 113.12, 113.20, 113.88, 114.25, 114.38, 114.89, 114.93, 114.99, 115.55, 117.28, 117.36, 117.62, 117.99, 118.85, 122.32, 123.75, 123.96, 124.15, 124.23, 124.40, 124.95, 125.25, 126.97, 127.01, 127.31, 127.48, 128.62, 129.00, 129.16, 129.75, 130.75, 130.81, 131.26, 131.93, 131.98, 132.23, 132.31, 133.27, 133.39, 135.37, 135.83, 138.23, 139.65, 139.80, 141.91, 142.77, 144.10, 144.15, 144.23, 145.04, 148.38, 148.55, 151.19, 152.42, 152.76, 153.18, 156.62, 156.71, 159.52, 159.73, 163.07.
HRMS (ESI): m/z: 1769.8463 (calcd. $[M+2Na]^{2+}$ = 1769.8470).

Polymer Synthesis

Scheme S5. Synthesis scheme of the polyurethane-urea into which **RotAnBP** is incorporated.

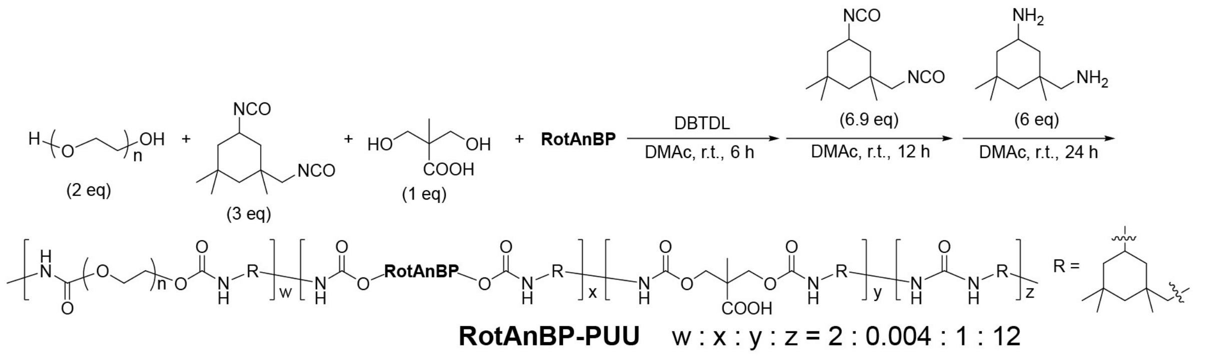

Fluorescence Spectra of An, BP, AnBP, and RotAnBP with Excitation at 590 nm

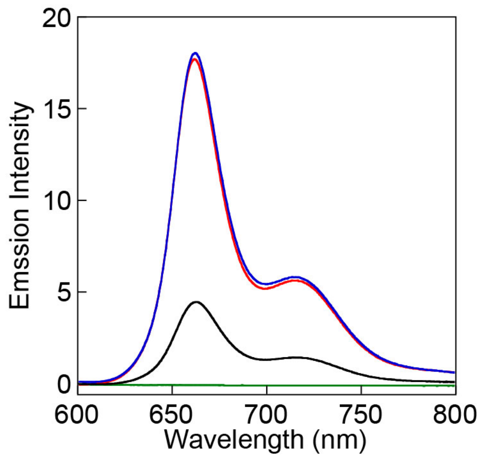

Figure S1. Fluorescence spectra of **An** (green), **BP** (red), **AnBP** (blue), and **RotAnBP** (black) in THF/methanol (1:4, v/v) solutions ($c = 1 \times 10^{-5}$ M) with excitation at 590 nm at r.t.

Calculation of Spectral Overlap Integral and Förster distance

Using equations (1) and (2), the spectral overlap integral ($J(\lambda)$) between the donor fluorescence and the acceptor absorption, and the Förster distance (R_0) of the donor/acceptor pair were determined:[S4]

$$J(\lambda) = \int_0^{\infty} f(\lambda)\varepsilon(\lambda)\lambda^4 \mathrm{d}\lambda \qquad (1)$$

$$R_0{}^6 = \frac{9}{4(2\pi)^5} * \frac{2303}{N} * \kappa^2 \varphi_{\mathrm{D}} n^{-4} J(\lambda) \qquad (2)$$

Here, $f(\lambda)$ is the normalized fluorescence intensity of the 9,10-bis(phenylethynyl)anthracene donor, $\varepsilon(\lambda)$ is the extinction coefficient of the π-extended BODIPY acceptor, N is Avogadro's number, κ^2 is the orientation factor of 2/3, φ_{D} is the fluorescence quantum yield of the donor (0.91[S1]), n is the refractive index of the 1:4, v:v THF/methanol solution (estimated to be 1.345), and the constants preceding κ have an aggregate value of 8.79×10^{-11} M cm nm^2. Using these values, $J(\lambda)$ and R_0 were calculated to be 5.581×10^{15} M^{-1} cm^{-1} nm^4 and 67.1 Å, respectively.

The energy transfer efficiency (E) is described by equation (3), where r is the distance between the donor and acceptor molecules. Using the value of the Förster distance reported above, the energy transfer efficiency should be higher than 99% when r is below 31.2 Å.

$$E = \frac{R_0^6}{R_0^6 + r^6} \qquad (3)$$

Excitation Spectra of AnBP and RotAnBP in THF/methanol

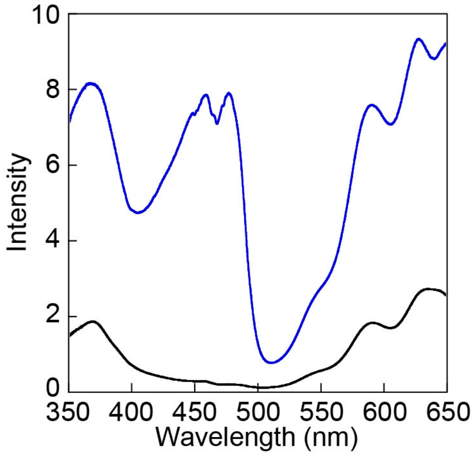

Figure S2. Excitation spectra of **AnBP** (blue) and **RotAnBP** (black) in THF/methanol (1:4, v/v) solutions ($c = 1 \times 10^{-5}$ M) recorded at an emission wavelength of 660 nm at r.t.

Time-Resolved Fluorescence Spectroscopy for BP, AnBP, and RotAnBP

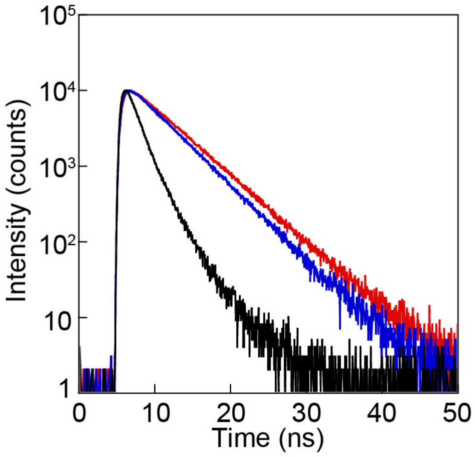

Figure S3. Emission decay profiles of **BP** (red), **AnBP** (blue), and **RotAnBP** (black) in THF/methanol (1:4, v/v) solutions ($c = 1 \times 10^{-5}$ M). The decay profiles were monitored at 660 nm with excitation at 470 nm.

Absorption and Fluorescence Spectra of RotAnBP-PUU

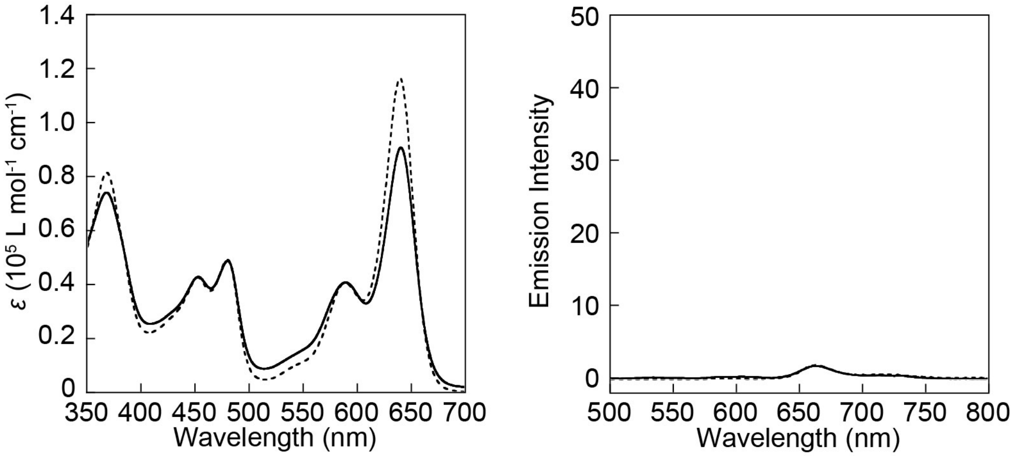

Figure S4. Absorption (left) and fluorescence (right) spectra of **RotAnBP** in THF/methanol (1:4, v/v) (dotted lines) and of **RotAnBP-PUU** in methanol (solid lines). The concentration of the **RotAnBP-PUU** solution was adjusted so that the absorbance at 490 nm matched the one of **RotAnBP**. The fluorescence spectra were recorded under the same conditions as the solution of **RotAnBP** shown in Figure 4.

^{1}H NMR Spectrum of RotAnBP-PUU

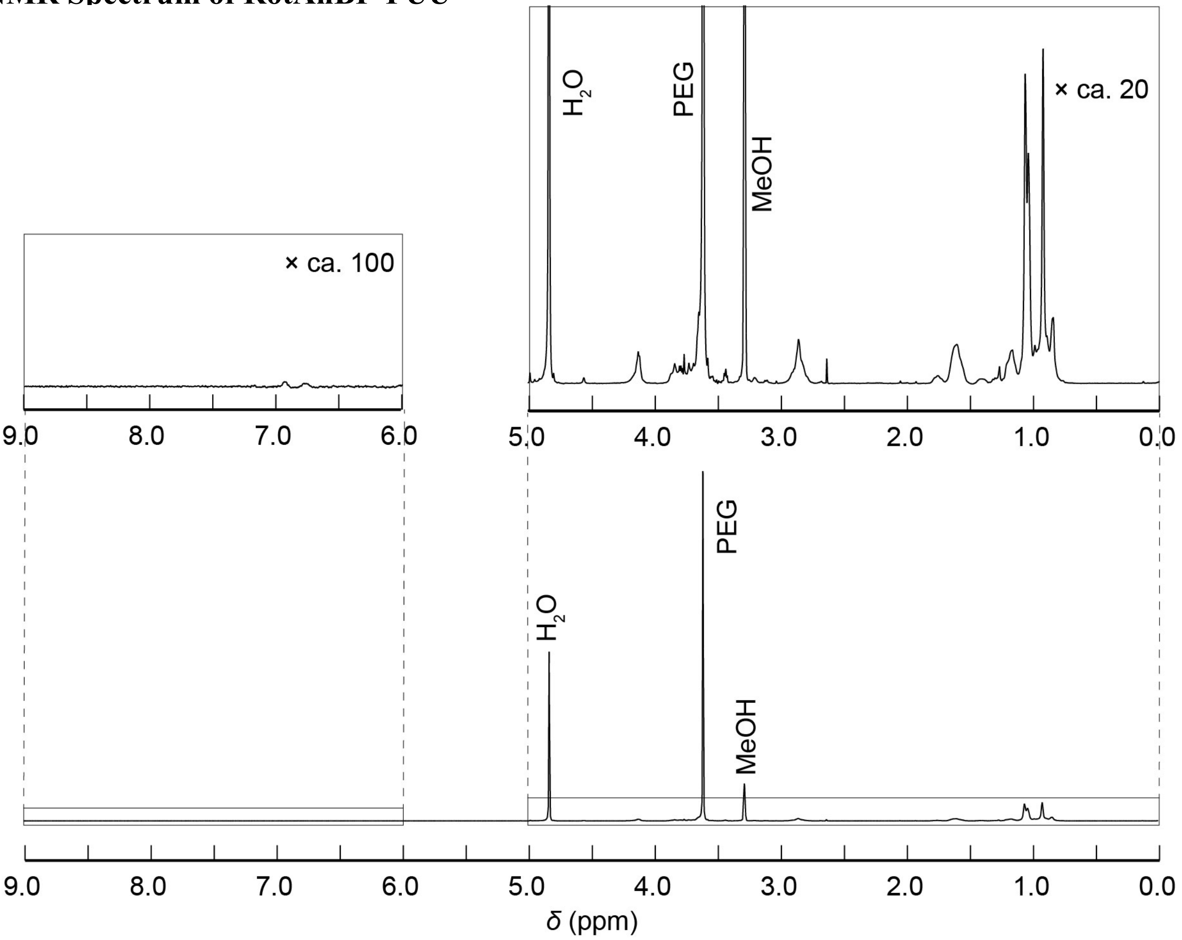

Figure S5. ^{1}H NMR spectrum of **RotAnBP-PUU** in methanol-d_4. No signals ascribed to the rotaxane mechanophore were observed due to the very low concentration. The spectrum was measured at r.t.

Thermal Properties of Dried RotAnBP-PUU Films

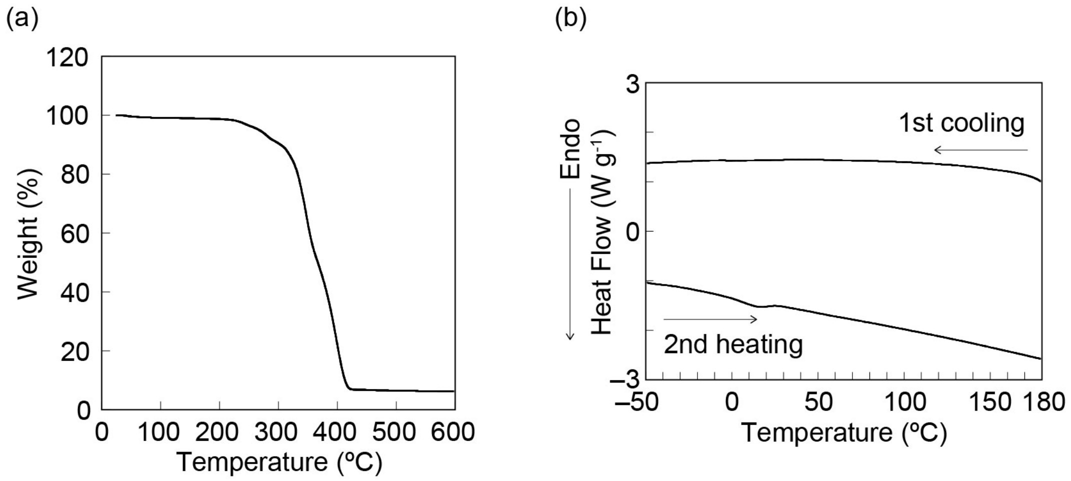

Figure S6. (a) TGA trace and (b) DSC traces of **RotAnBP-PUU**. The heating and cooling rates were 10 °C/min.

Mechanical Properties of Dried RotAnBP-PUU Films and RotAnBP-PUU Hydrogels

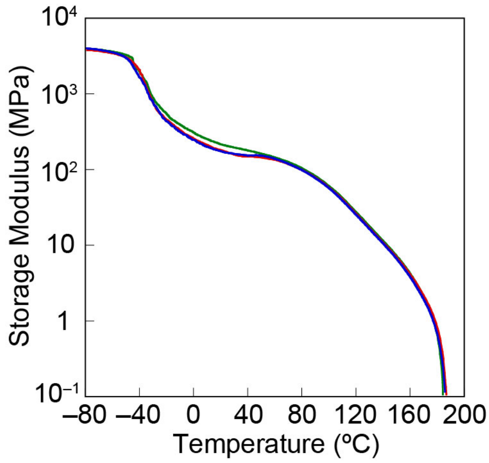

Figure S7. DMA traces of dried **RotAnBP-PUU** films. The graph shows data obtained from three different specimens. The tests were conducted under N_2 at a heating rate of 3 °C/min, a frequency of 1 Hz, and an amplitude of 15 μm.

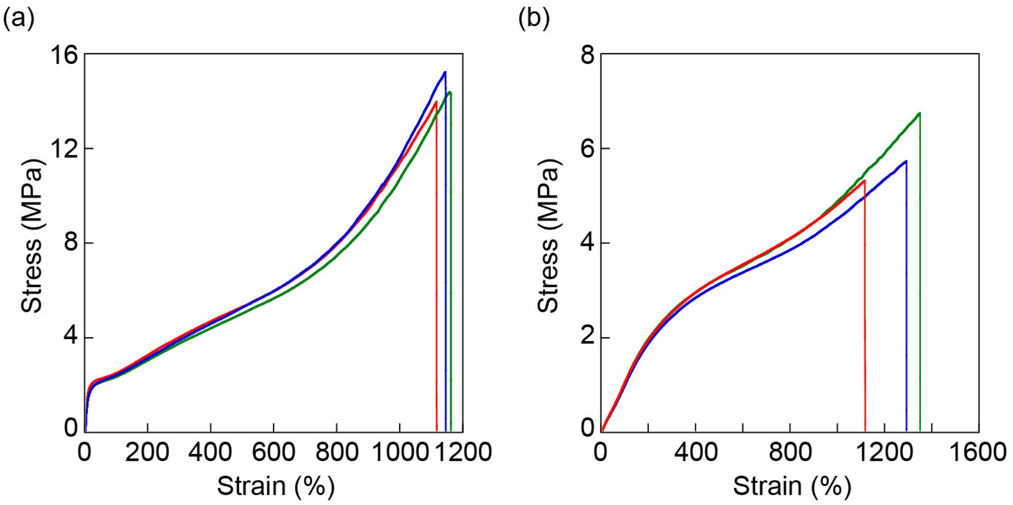

Figure S8. Strain-stress curves of (a) dried **RotAnBP-PUU** films and (b) **RotAnBP-PUU** hydrogels. Each graph shows data obtained from three different specimens. The experiments were conducted with a strain rate of 60 mm/min at r.t.

Table S1. Mechanical data of dried **RotAnBP-PUU** films and **RotAnBP-PUU** hydrogels extracted from tensile tests.[a)]

	Elongation at break	Stress at break	Young's modulus[b)]
Dried **RotAnBP-PUU** film	1142 ± 24 %	14.5 ± 0.8 MPa	29.5 ± 7.8 MPa
**RotAnBP-PUU** hydrogel	1254 ± 137 %	5.93 ± 0.82 MPa	1.19 ± 0.04 MPa

a) All data were extracted from the strain-stress curves shown in Figure S8 and represent averages of 3 measurements ± standard derivation. b) The Young's moduli were derived from the slopes of the strain-stress curves in the strain regime between 1.5–2%.

Change of Water Content upon Swelling the Dried RotAnBP-PUU Film

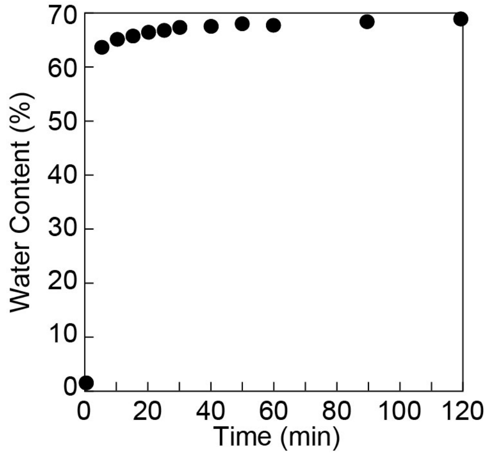

Figure S9. Water content of a **RotAnBP-PUU** film as a function of immersion time in an excess amount of deionized water at r.t. The water content was calculated by: Water content (%) = 100 × ($M - M_0$)/M, where M is the weight of the film after swelling, and M_0 is the initial weight of the dried film.

Decrease of Water Content in RotAnBP-PUU Hydrogel

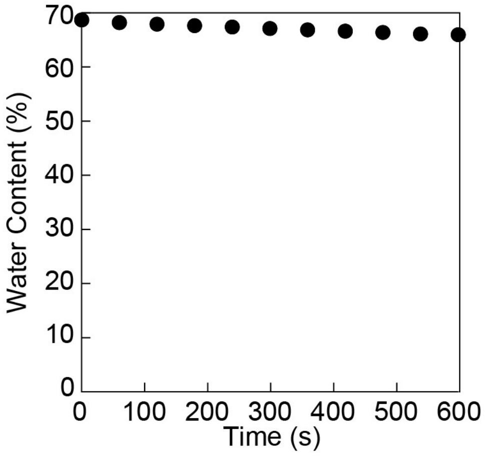

Figure S10. Change of the water content in the **RotAnBP-PUU** hydrogel as a function of the time under ambient conditions.

Images of RotAnBP-PUU Hydrogel and Dried RotAnBP-PUU Films upon Uniaxial Deformation

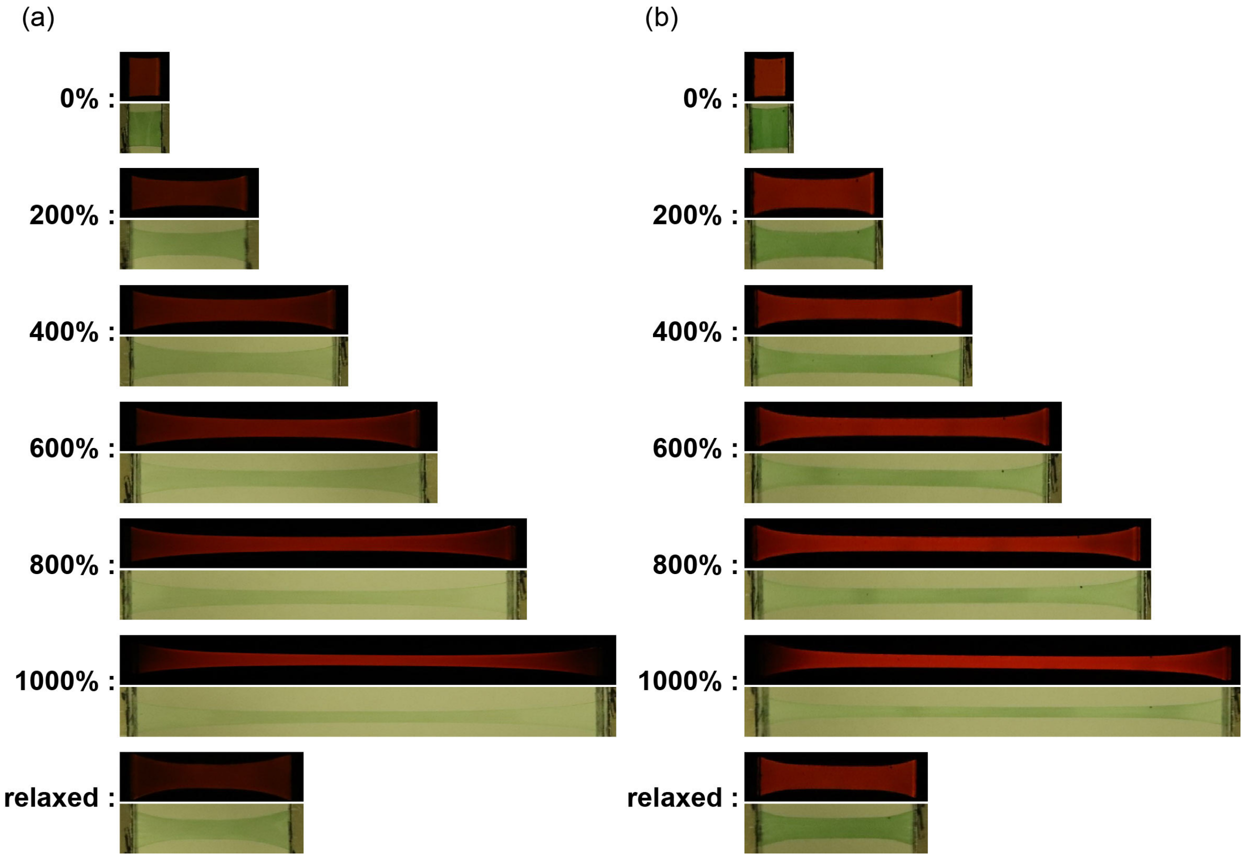

Figure S11. Pictures of (a) **RotAnBP-PUU** hydrogel and (b) dried **RotAnBP-PUU** films during uniaxial deformation test. In each strained state, the top picture displays the fluorescence of the hydrogel; these images were taken in the dark upon excitation at 490 nm, and a long pass filter cutting below 550 nm was placed in front of the camera. The bottom picture shown for each strain was taken under ambient illumination. All images were taken under the same ambient conditions.

Changes in the Fluorescence Intensity of RotAnBP-PUU Hydrogel upon Relaxation

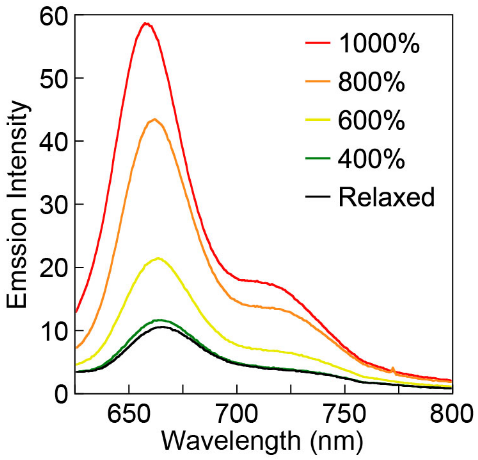

Figure S12. Fluorescence spectra of **RotAnBP-PUU** hydrogel recorded upon relaxation from the maximum strain of 1000% in the first cycle. The fluorescence spectra were recorded at r.t. with λ_{ex} = 490 nm.

Changes in the Fluorescence Intensity of RotAnBP-PUU Hydrogel upon Excitation at 590 nm

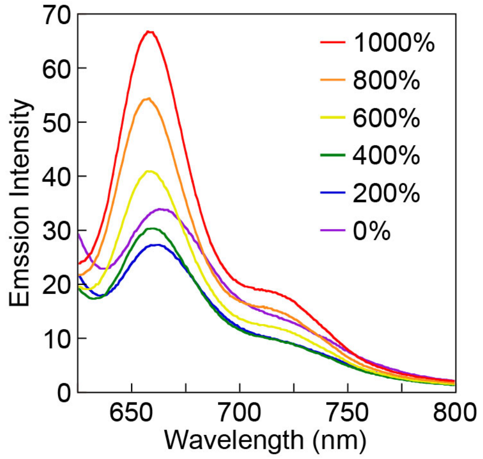

Figure S13. Fluorescence spectra of **RotAnBP-PUU** hydrogel upon stretching the samples to the strains indicated in the first cycle. The fluorescence spectra were recorded at r.t. with λ_{ex} = 590 nm.

Examination of the Penetration of the Red Fluorescence Emitted by RotAnBP-PUU Hydrogels

Preparation of RotAnBP-PUU Hydrogels Based on a Second Batch of RotAnBP-PUU. In this experiment, **RotAnBP-PUU** hydrogels were prepared using a second batch of **RotAnBP-PUU** that was synthesized according to the procedure and reaction scheme described above (Scheme S5). The mechanical response of the newly prepared hydrogel (Figure S14) is similar to that of the hydrogels used in other experiments (Figure S8).

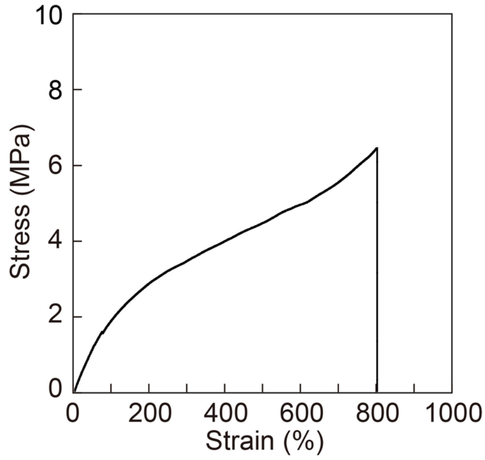

Figure S14. Stress-strain curve of a **RotAnBP-PUU** hydrogel based on a second batch of **RotAnBP-PUU**. The experiment was conducted with a strain rate of 60 mm/min at r.t.

Reference Polyurethane Films in which a Rotaxane Mechanophore Showing On/Off Switching of Green Emission Was Incorporated. For reference purposes, a polyurethane that we reported as **Rot2PU** in a previous paper[S5] and which contained the green-light emitting rotaxane **Rot 2** was employed. The molecular structures of **Rot2** and **Rot2PU** are shown in Figure S15.

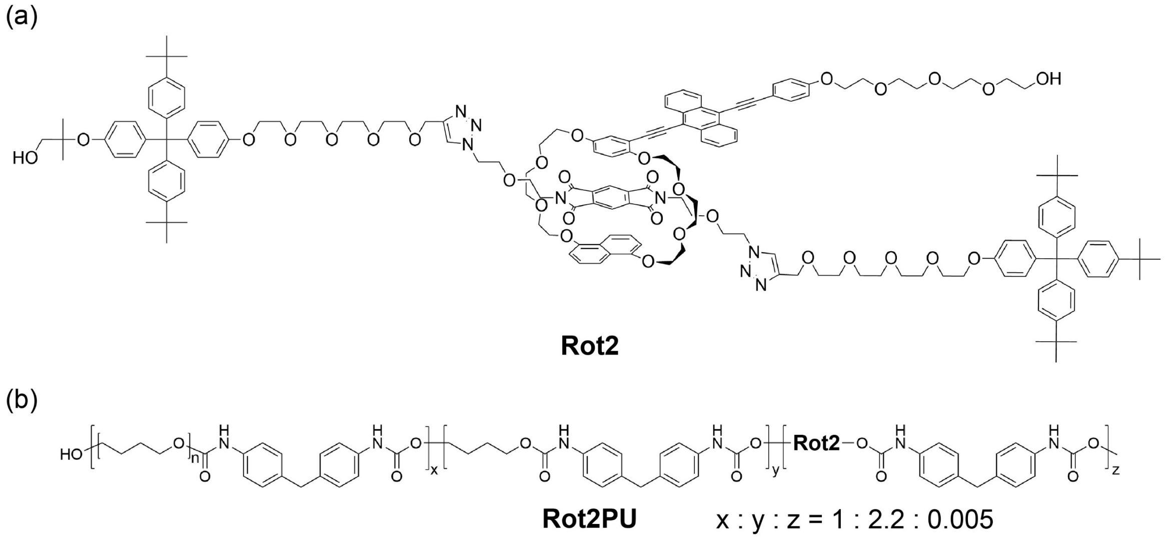

Figure S15. Molecular structures of (a) **Rot2** and (b) **Rot2PU**.

Preparation of Red PU Films without Any Mechanophores. The red-colored PU films were prepared by physically doping sudan III (Figure S16a and S16b) into the reference polyurethane without any mechanophores. Thus, PU (300 mg, M_n = 121 kg/mol, PDI = 2.04) synthesized according to the method that we previously reported[S1] and 10 mg of sudan III were dissolved in THF (5 mL) and the solution was divided between two square poly(tetrafluoroethylene) molds (51 × 51 × 5.0 mm). The molds were placed under an inverted funnel to control the evaporation of the solvent. The solvent was evaporated over the course of 4 h under ambient conditions and the resulting films were further dried in vacuo at r.t. for 12 h. The red films thus obtained were smooth and opaque with a thickness of 60–80 μm. A characteristic stress-strain curve of the films thus obtained is shown in Figure S16c.

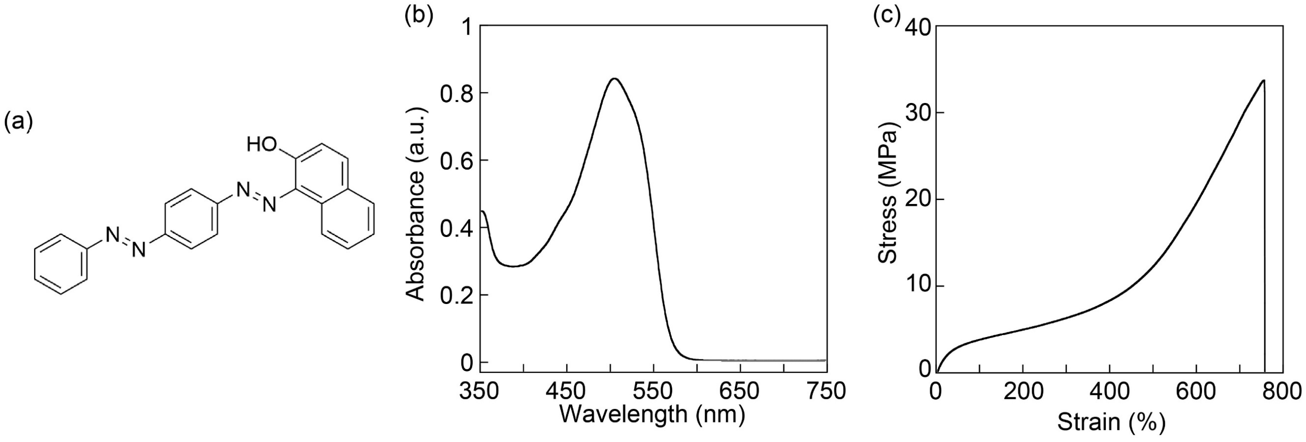

Figure S16. (a) Molecular structure of sudan III. (b) Absorption spectrum of sudan III in THF solution. (c) Stress-strain curve of a PU film containing sudan III. The experiment was conducted with a strain rate of 120 mm/min at r.t.

Changes in Fluorescence Spectra of Hydrogels and Films Overlaid with the Red Films upon Stretching. Tensile tests were performed for samples of **RotAnBP-PUU** hydrogel or **Rot2PU** film overlaid without and with the red films (Figure S17). While stretching tests, the excitation light was irradiated to the center of **RotAnBP-PUU** hydrogel or **Rot2PU** film from one optical fiber connected to the light source, and the fluorescence of the **RotAnBP-PUU** hydrogel or **Rot2PU** film passing through the red PU film was monitored by the other optical fiber connected to the detector.

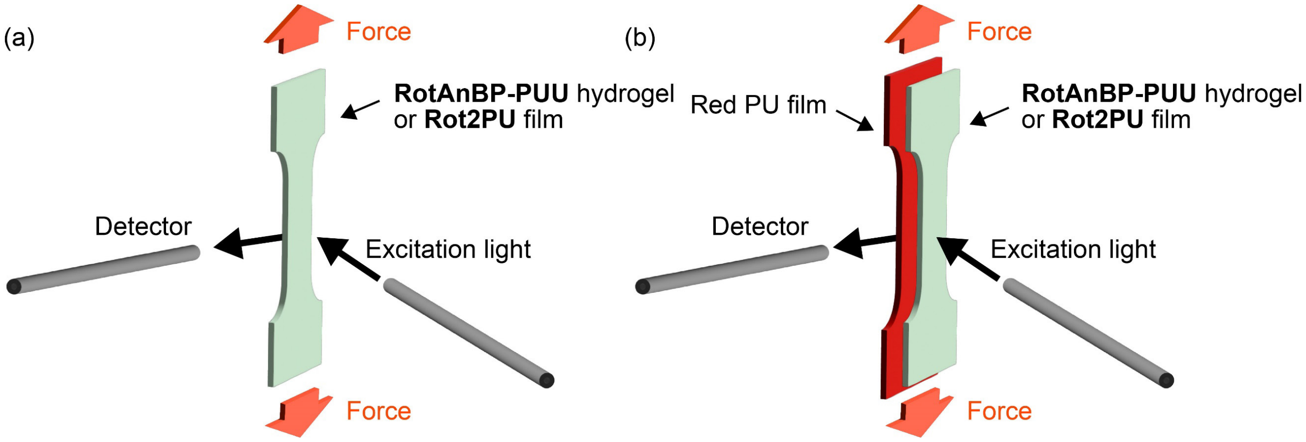

Figure S17. Schematic illustration of the setup used to examine the permeability of red fluorescence exhibited by **RotAnBP-PUU** hydrogels and green fluorescence of **Rot2PU** film overlaid (a) without and (b) with the red films.

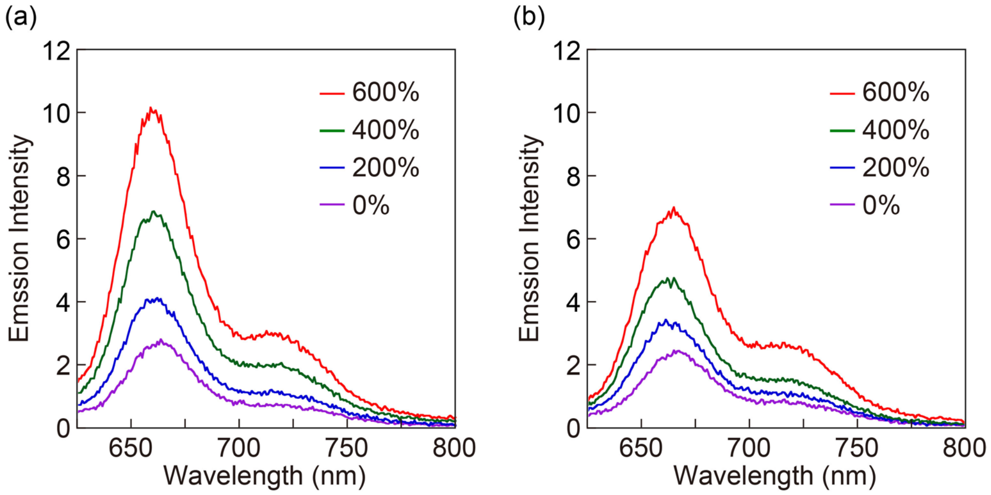

Figure S18. Fluorescence spectra of (a) **RotAnBP-PUU** hydrogel and (b) **RotAnBP-PUU** hydrogel overlaid with the red film upon stretching the samples to the strains indicated in the first cycle. The fluorescence spectra were recorded at r.t. with λ_{ex} = 490 nm.

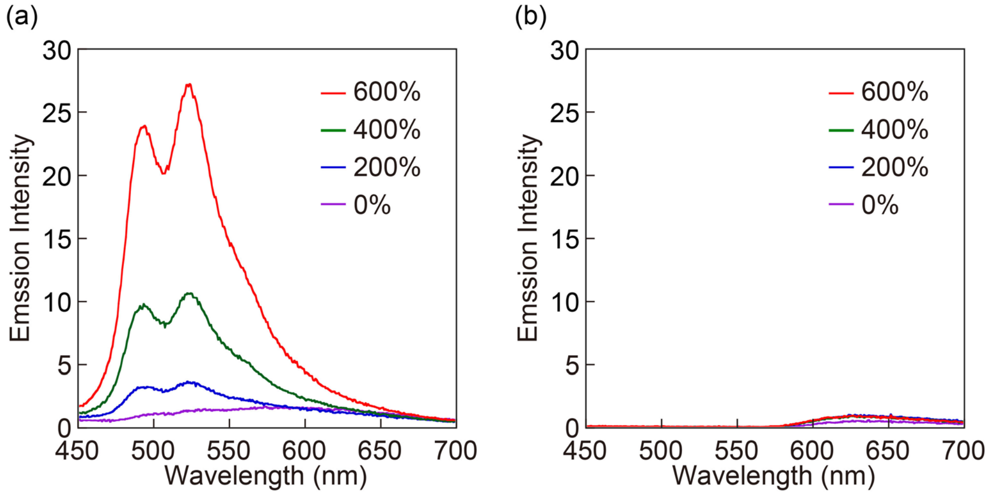

Figure S19. Fluorescence spectra of (a) **Rot2PU** and (b) **Rot2PU** overlaid with the red film upon stretching the samples to the strains indicated in the first cycle. The fluorescence spectra were recorded at r.t. with λ_{ex} = 365 nm.

References

S1. Sagara, Y.; Karman, M.; Seki, A.; Pannipara, M.; Tamaoki, N.; Weder, C. Rotaxane-Based Mechanophores Enable Polymers with Mechanically Switchable White Photoluminescence. *ACS Cent. Sci.* **2019**, *5*, 874−881.

S2. Zhang C.; Zhao J.; Wu S.; Wang Z.; Wu W.; Ma J.; Guo S.; Huang L. Intramolecular RET Enhanced Visible Light-Absorbing Bodipy Organic Triplet Photosensitizers and Application in Photooxidation and Triplet–Triplet Annihilation Upconversion. *J. Am. Chem. Soc.* **2013**, *135*, 10566–10578.

S3. Sagara, Y.; Karman, M.; Verde-Sesto, E.; Matsuo, K.; Kim, Y.; Tamaoki, N.; Weder, C. Rotaxanes as Mechanochromic Fluorescent Force Transducers in Polymers. *J. Am. Chem. Soc.* **2018**, *140*, 1584–1587.

S4. Patterson, G. H.; Piston D. W.; Barisas B. G. Förster Distances between Green Fluorescent Protein Pairs. *Anal. Biochem.* **2000**, *284*, 438–440.

S5. Muramatsu, T.; Okado, Y.; Traeger, H.; Schrettl, S.; Tamaoki, N.; Weder, C.; Sagara, Y. Rotaxane-Based Dual Function Mechanophores Exhibiting Reversible and Irreversible Responses. *J. Am. Chem. Soc.* **2021**, *143*, 9884–9892.

Supporting Movies

Movies S1 and S2. Representative movies of the mechanoresponsive luminescent behavior upon cyclic stretching of a **RotAnBP-PUU** hydrogel and a dried **RotAnBP-PUU** film.
The movies were taken in the dark under excitation at 490 nm and a long pass filter cutting below 550 nm was placed in front of the camera.

NMR Spectra

(a)

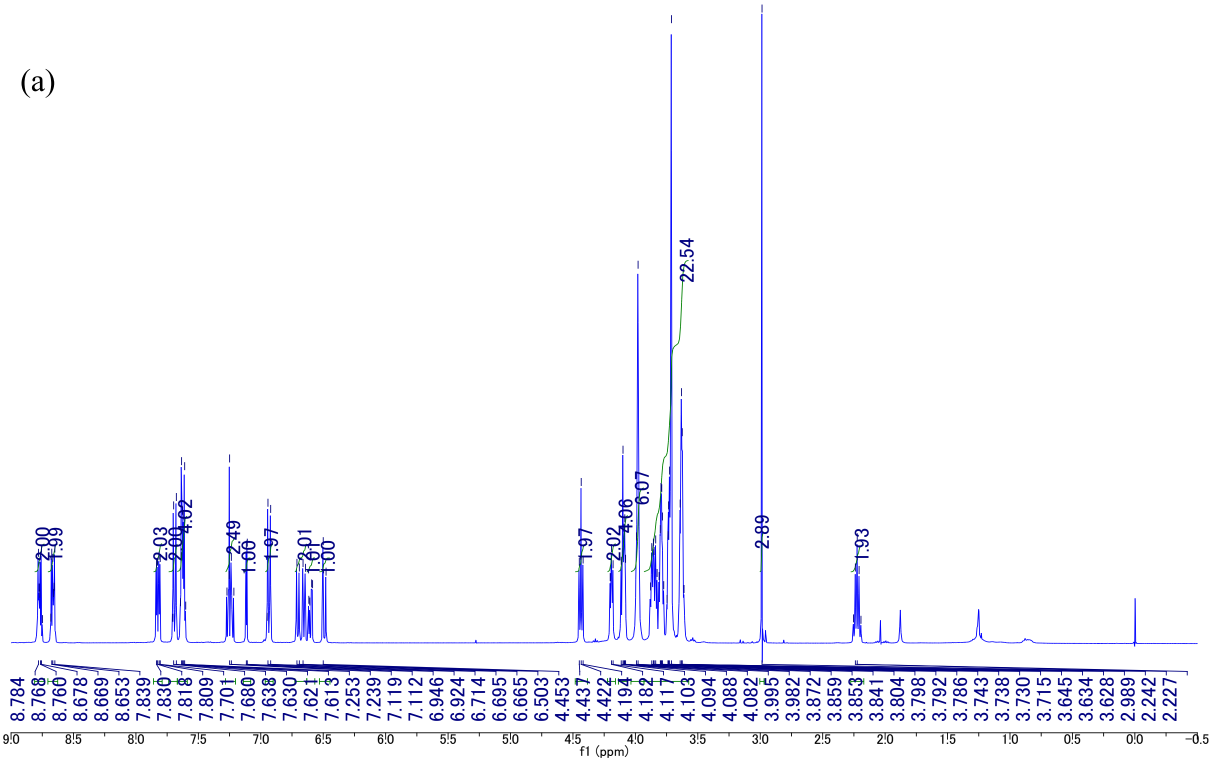

(b)

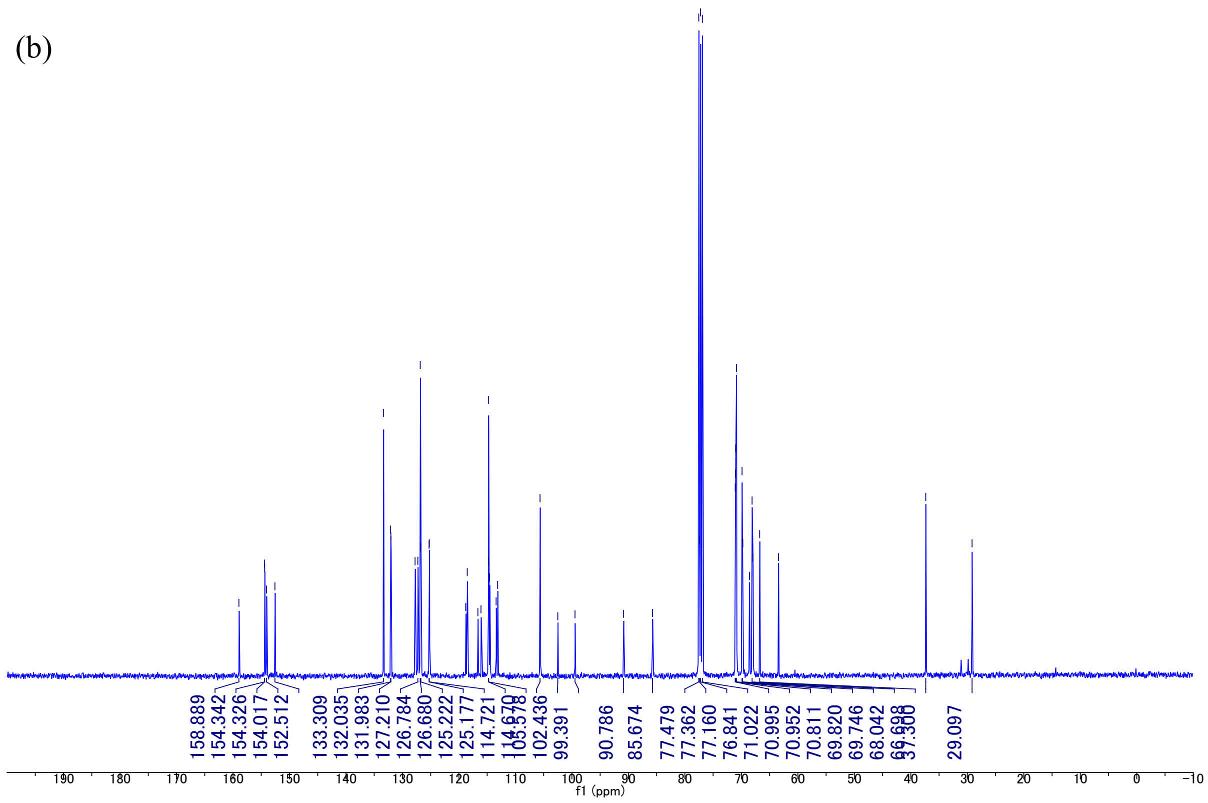

Figure S20. (a) ^{1}H NMR and (b) ^{13}C NMR spectra of compound **1**.

(a)

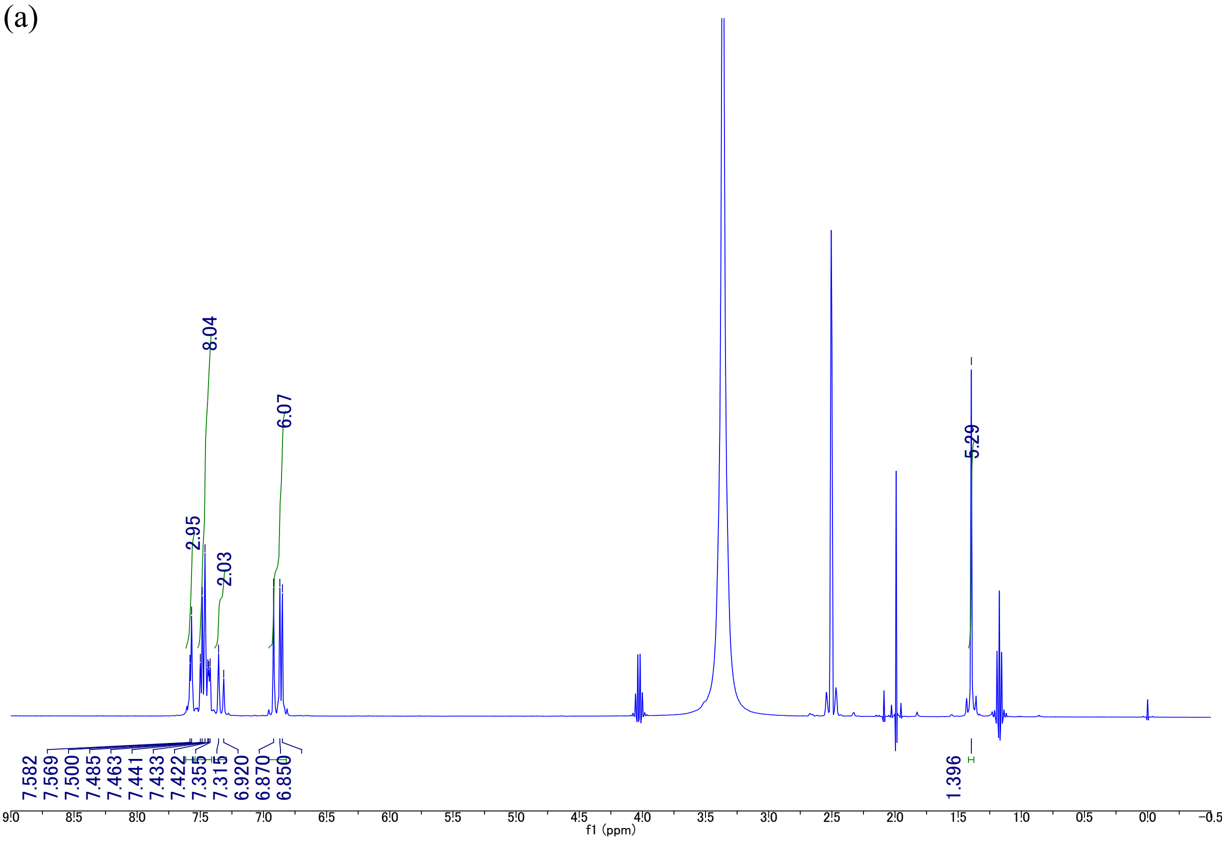

(b)

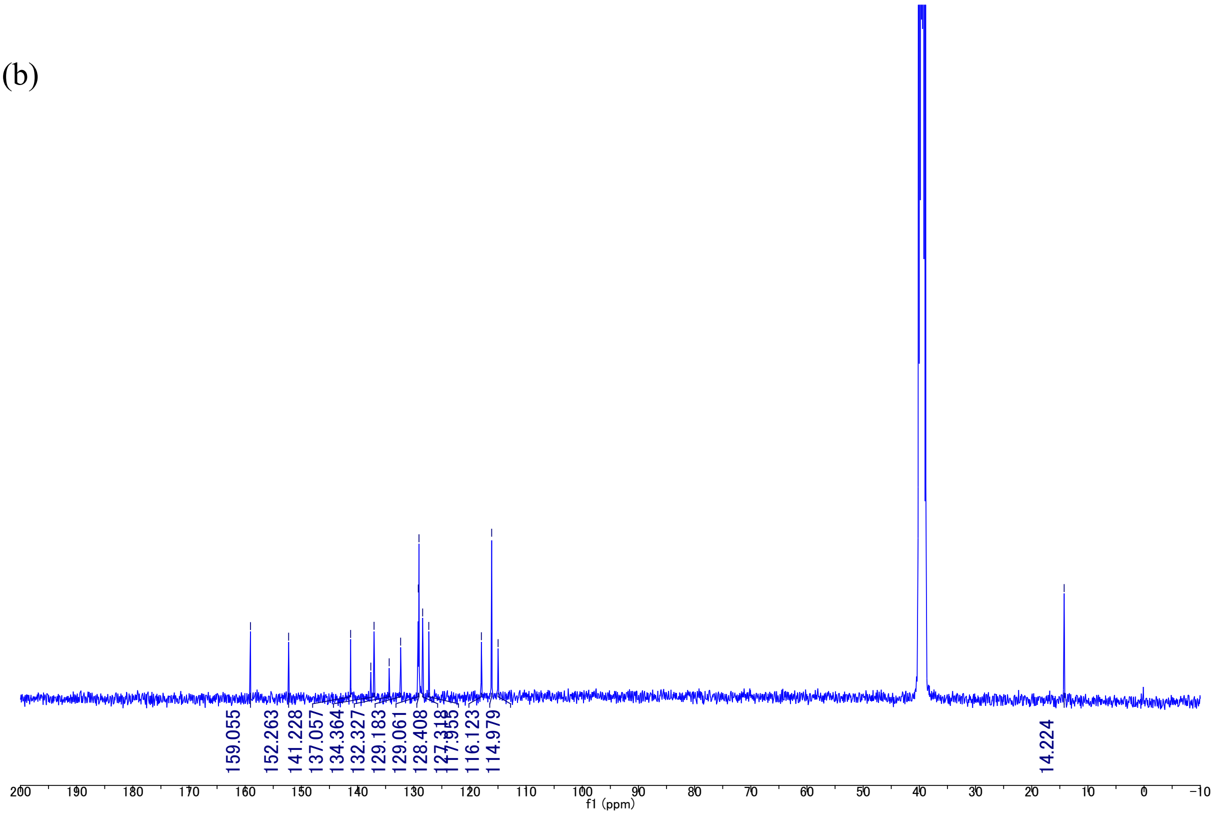

Figure S21. (a) ^{1}H NMR and (b) ^{13}C NMR spectra of compound **2**.

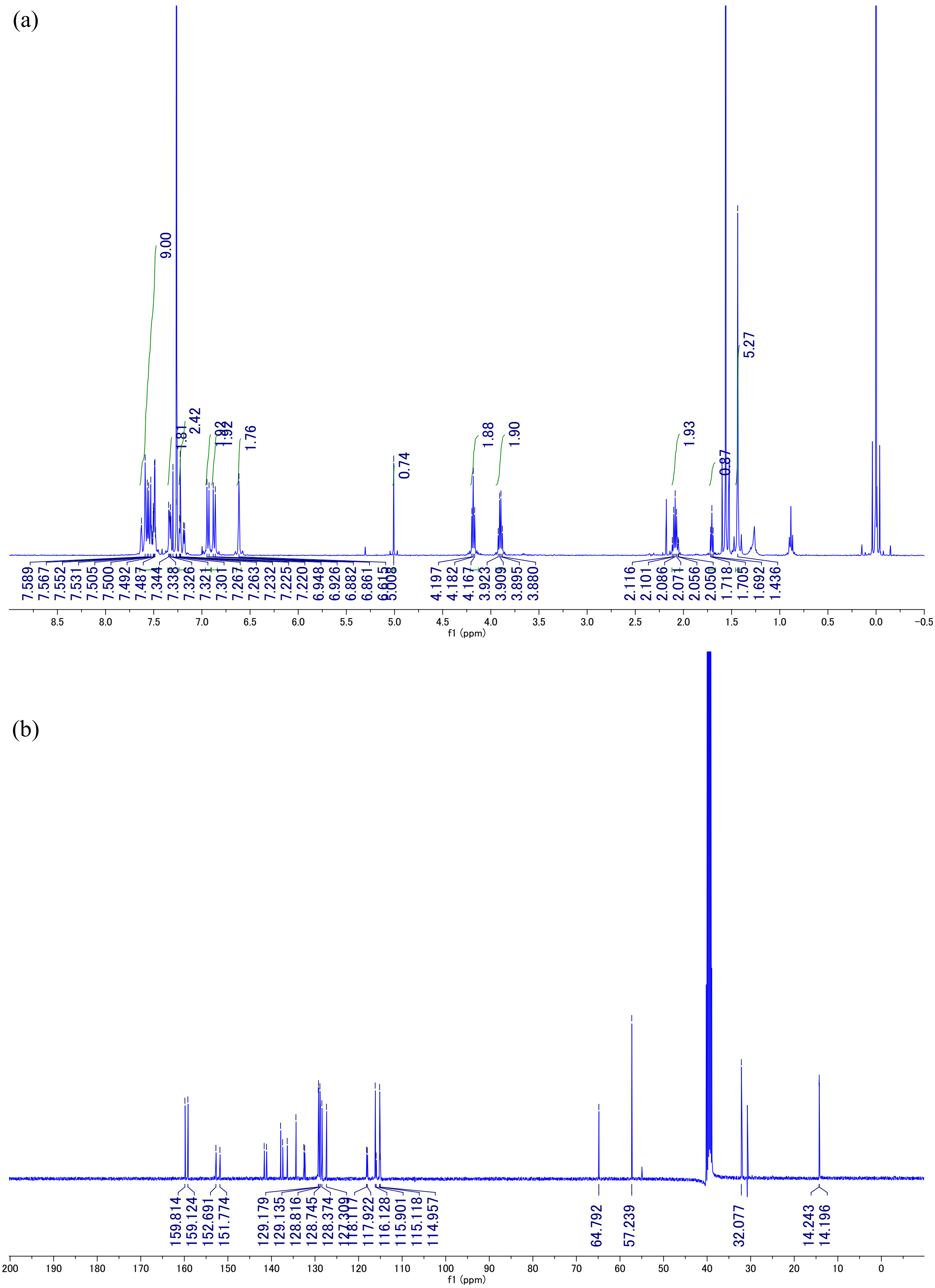

Figure S22. (a) ^{1}H NMR and (b) ^{13}C NMR spectra of compound **3**.

(a)

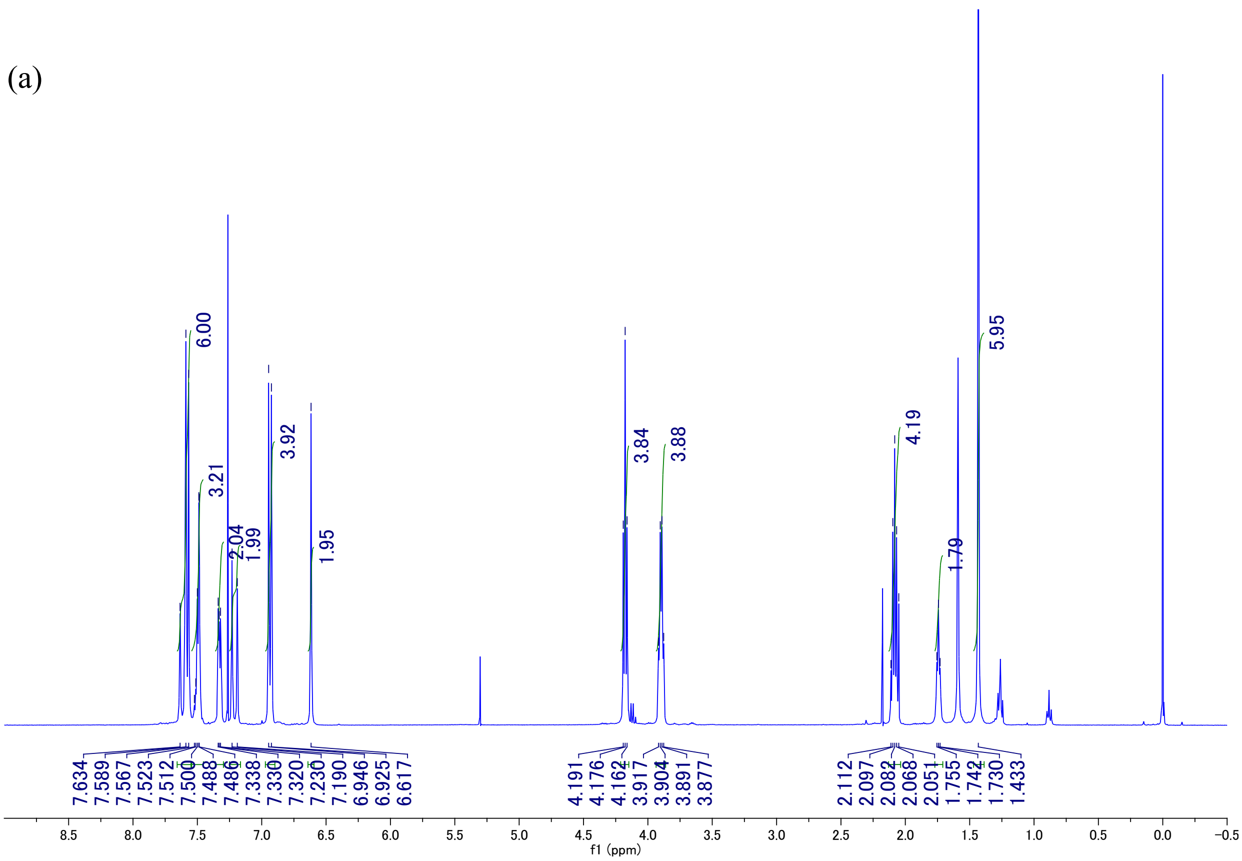

(b)

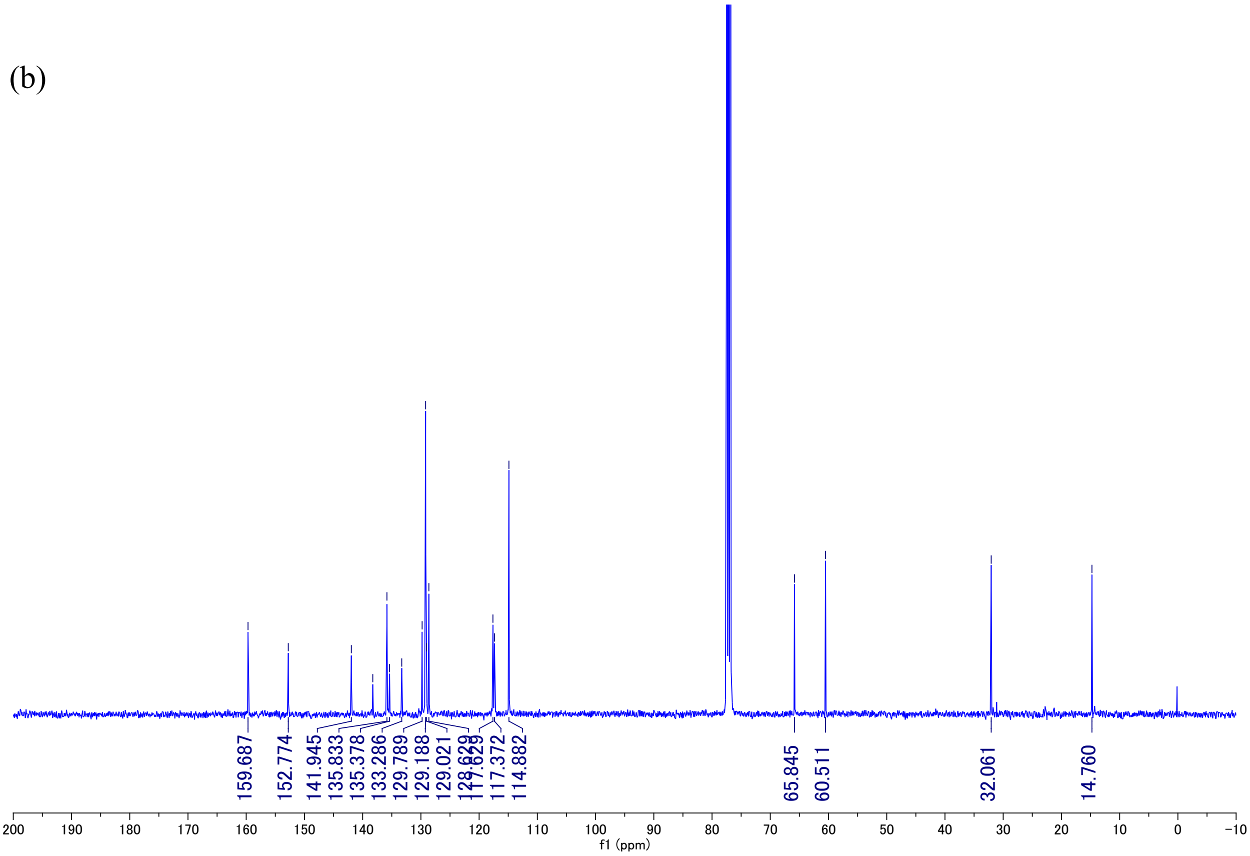

Figure S23. (a) ^{1}H NMR and (b) ^{13}C NMR spectra of compound **BP**.

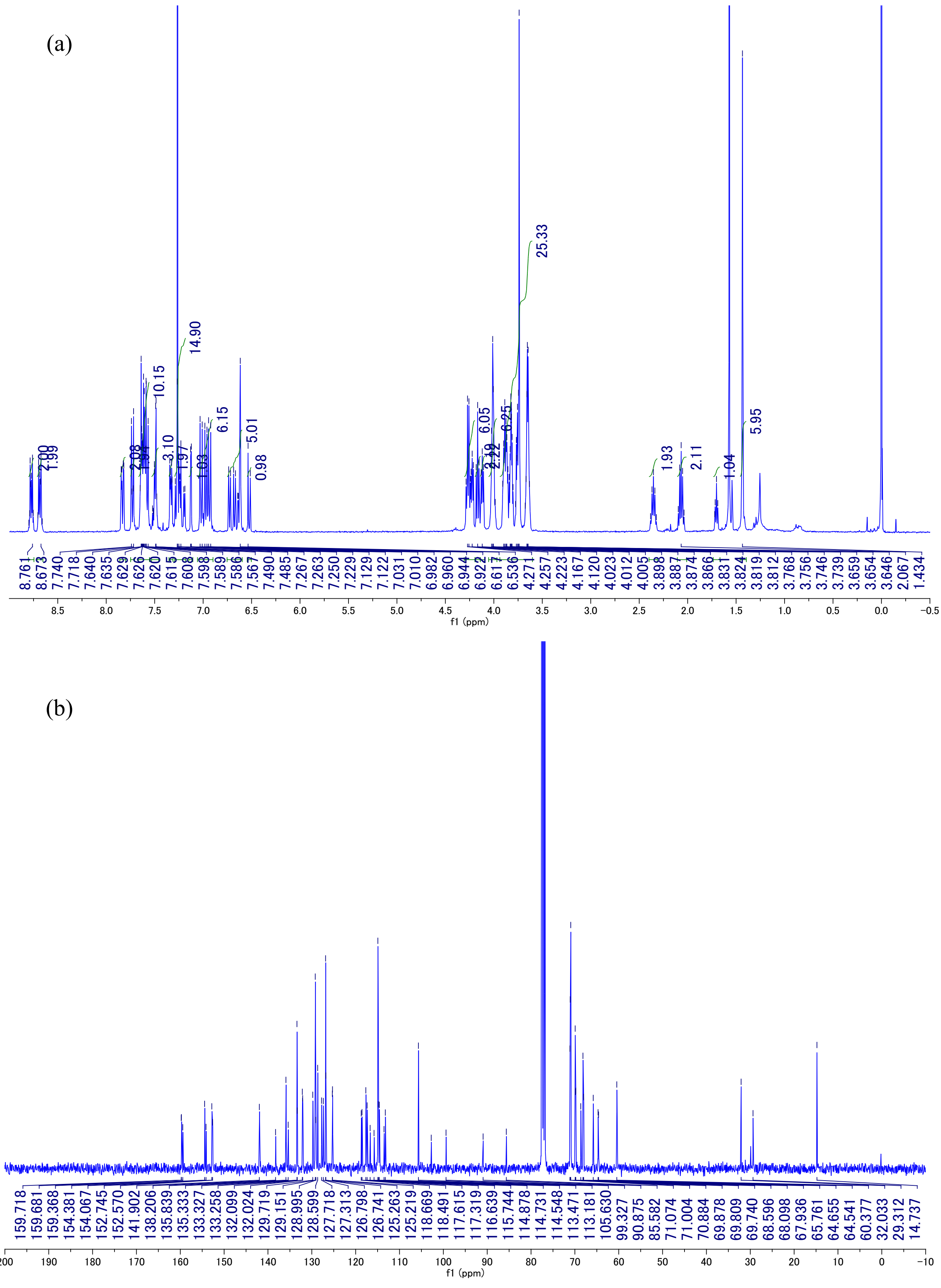

Figure S24. (a) ^{1}H NMR and (b) ^{13}C NMR spectra of compound **AnBP**.

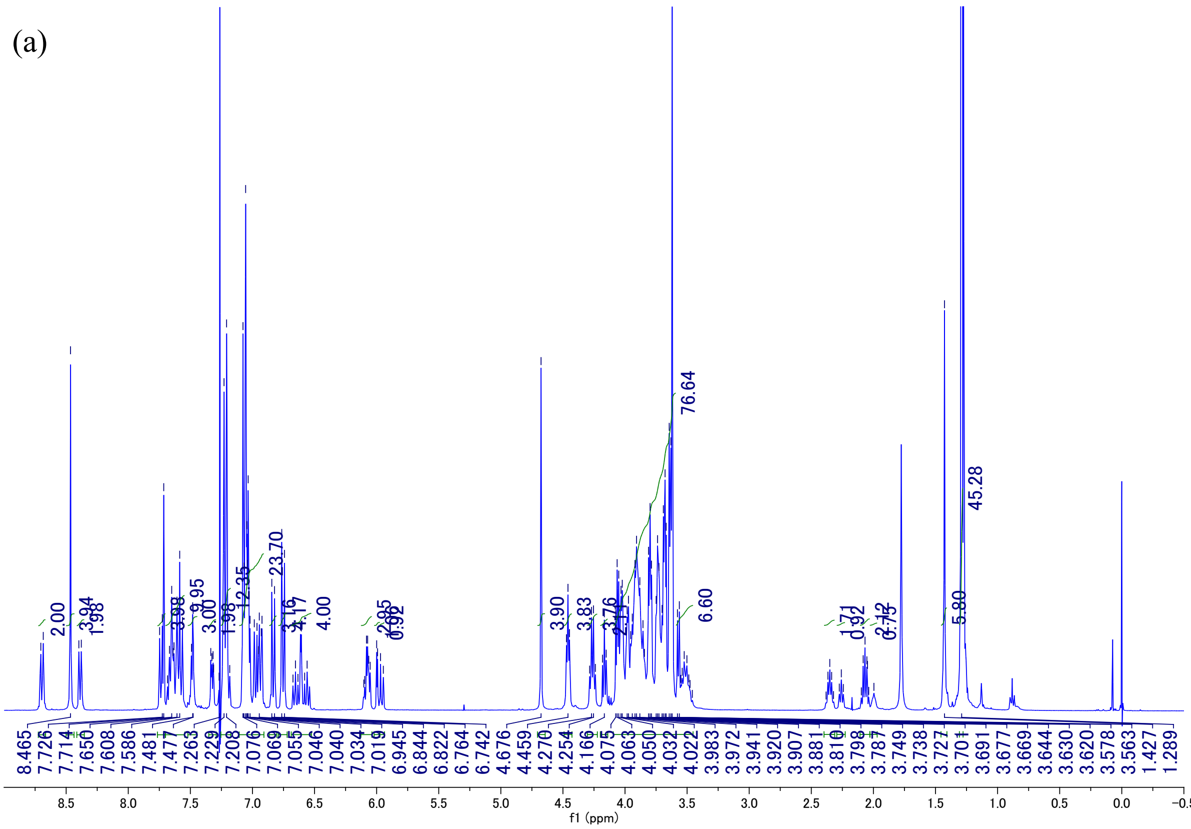

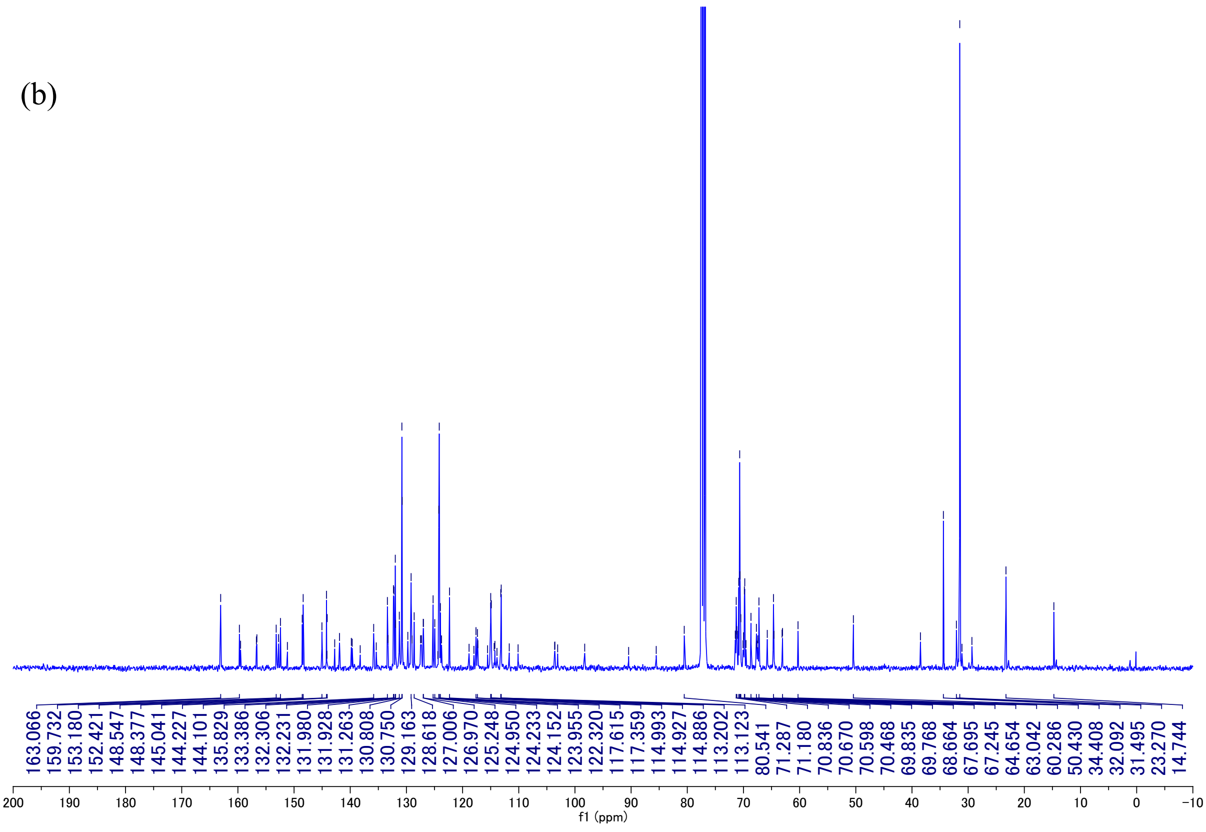

Figure S25. (a) ^{1}H NMR and (b) ^{13}C NMR spectra of compound **RotAnBP**.
